# Influence of Peripheral Alkyl Groups on Junction Configurations in Single-Molecule Electronics

**DOI:** 10.1021/acs.jpcc.3c06970

**Published:** 2024-01-16

**Authors:** Luca Ornago, Patrick Zwick, Sebastiaan van der Poel, Thomas Brandl, Maria El Abbassi, Mickael L. Perrin, Diana Dulić, Herre S. J. van der Zant, Marcel Mayor

**Affiliations:** † Kavli Institute of Nanoscience, Delft University of Technology, Lorentzweg 1, 2628 CJ Delft, The Netherlands; ‡ Department of Chemistry, University of Basel, St. Johanns-Ring 19, 4056 Basel, Switzerland; § Transport at Nanoscale Interfaces Laboratory, Empa, Swiss Federal Laboratories for Materials Science and Technology, 8600 Dübendorf, Switzerland; ∥ Department of Information Technology and Electrical Engineering, ETH Zürich, 8092 Zürich, Switzerland; ⊥ Quantum Center, ETH Zürich, 8093 Zürich, Switzerland; # Department of Physics and Department of Electrical Engineering, Faculty of Physical and Mathematical Sciences, University of Chile, Avenida Blanco Encalada 2008, Santiago 8330015, Chile; ∇ Institute for Nanotechnology (INT), Karlsruhe Institute of Technology (KIT), P.O. Box 3640, 76021 Karlsruhe, Germany; ○ Lehn Institute of Functional Materials (LIFM), School of Chemistry, Sun Yat-Sen University (SYSU), Guangzhou 510275, China

## Abstract

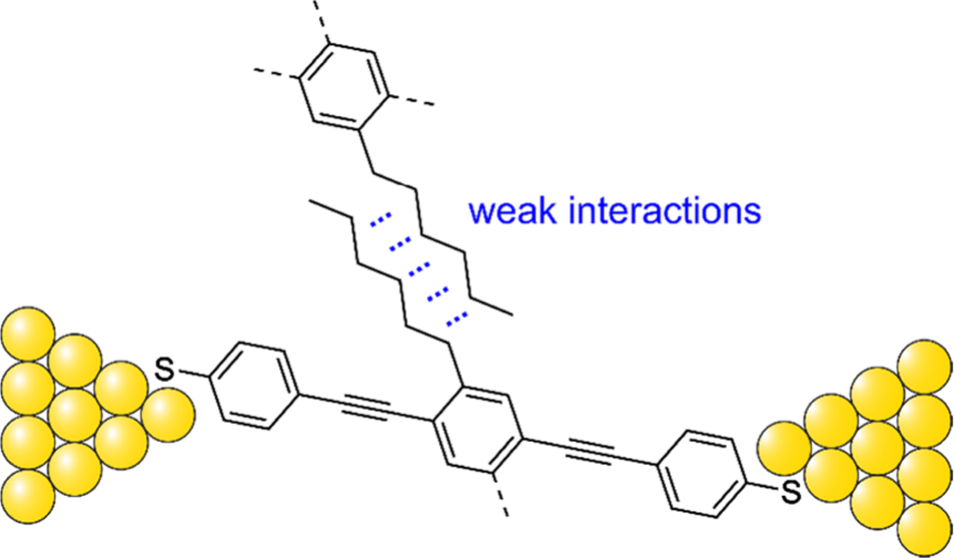

The addition of a lateral alkyl chain is a well-known strategy to reduce π-stacked ensembles of molecules in solution, with the intention to minimize the interactions between the molecules’ backbones. In this paper, we study whether this concept generalizes to single-molecule junctions by using a combination of mechanically controllable break junction (MCBJ) measurements and clustering-based data analysis with two small series of model compounds decorated with various bulky groups. The systematic study suggests that introducing alkyl side chains also favors the formation of electrode-molecule configurations that are not observed in their absence, thereby inducing broadening of the conductance peak in the one-dimensional histograms. Thus, the introduction of alkyl chains in aromatic compounds for molecular electronics must be carefully designed and optimized for the specific purpose, balancing between increased solubility and the possibility of additional junction configurations.

## Introduction

Molecules are the tiniest objects that can perform specific functions arising from their structure (i.e., the nature of the individual atoms of which they are made and the connectivity and spatial arrangement of those). Joint efforts by chemists, physicists, and theoreticians in the interdisciplinary field of molecular electronics have led to numerous charge transport studies on single molecules, captured between two nanoelectrodes, as shown by literature reviews.^1−7^ A key challenge toward the use of single molecules in future devices is the robustness of the formed molecular junction, which is typically characterized statistically by repeating conductance measurements. Often, multiple conductance values are measured, corresponding to different configurations that the molecule can adopt when connected to the electrodes. With the development of unsupervised clustering algorithms, the analysis of these different configurations has been greatly improved, even allowing for the identification of junction configuration with a low occurrence.^8−11^ One of the mechanisms discussed in the literature that introduces this complication is the formation of π–π stacked ensembles of molecules within the junction.^12−14^ To achieve the goal of well-defined and reproducible molecular junctions, it is thus crucial to comprehend and control this type of intermolecular interactions.

In wet chemistry, bulky side chains are often used to prevent π–π stacking with the goal of increasing solubility.^15,16^ The isolation of the π-backbone in a molecular junction supposedly decreases the conductance spread by insulating the molecule from other molecules and undesired electron injection from the electrodes.^17,18^ We recently reported on an increase of the number of junctions performing their designed function by increasing the distance between the electrodes and the functional subunit, as well as shielding the latter by adding branched alkane units.^19^ Additionally, it has been reported that the addition of alkane side groups can reduce the density of molecules at the electrode tip, thereby decreasing the probability of the in situ formation of junctions consisting of two or three molecules in parallel.^20^

In this study, we analyze whether the concept of reducing intermolecular end-environmental interactions by the addition of alkyl side groups can be generalized to metal-molecule-metal junctions. Similar to the solubility of aromatic compounds in wet chemistry, more alkane chains are supposed to lead to a better isolation of the molecular backbone. As established models in molecular electronics, we consider a series of oligophenylethynylenes (OPEs) and porphyrins to benchmark the alkyl chains’ effects.^21−24^ Series of both model compounds, decorated with different alkyl chains at different positions on the molecules, were studied in a mechanically controlled break junction (MCBJ) setup. The resulting transport data were analyzed by an unsupervised clustering algorithm to extract different molecular behaviors, also known as molecular classes.^9^ We correlate the structural differences of the molecules with the different classes and, therefore, the probability observed in the charge transport measurements.

## Experimental Section

**Figure 1:**
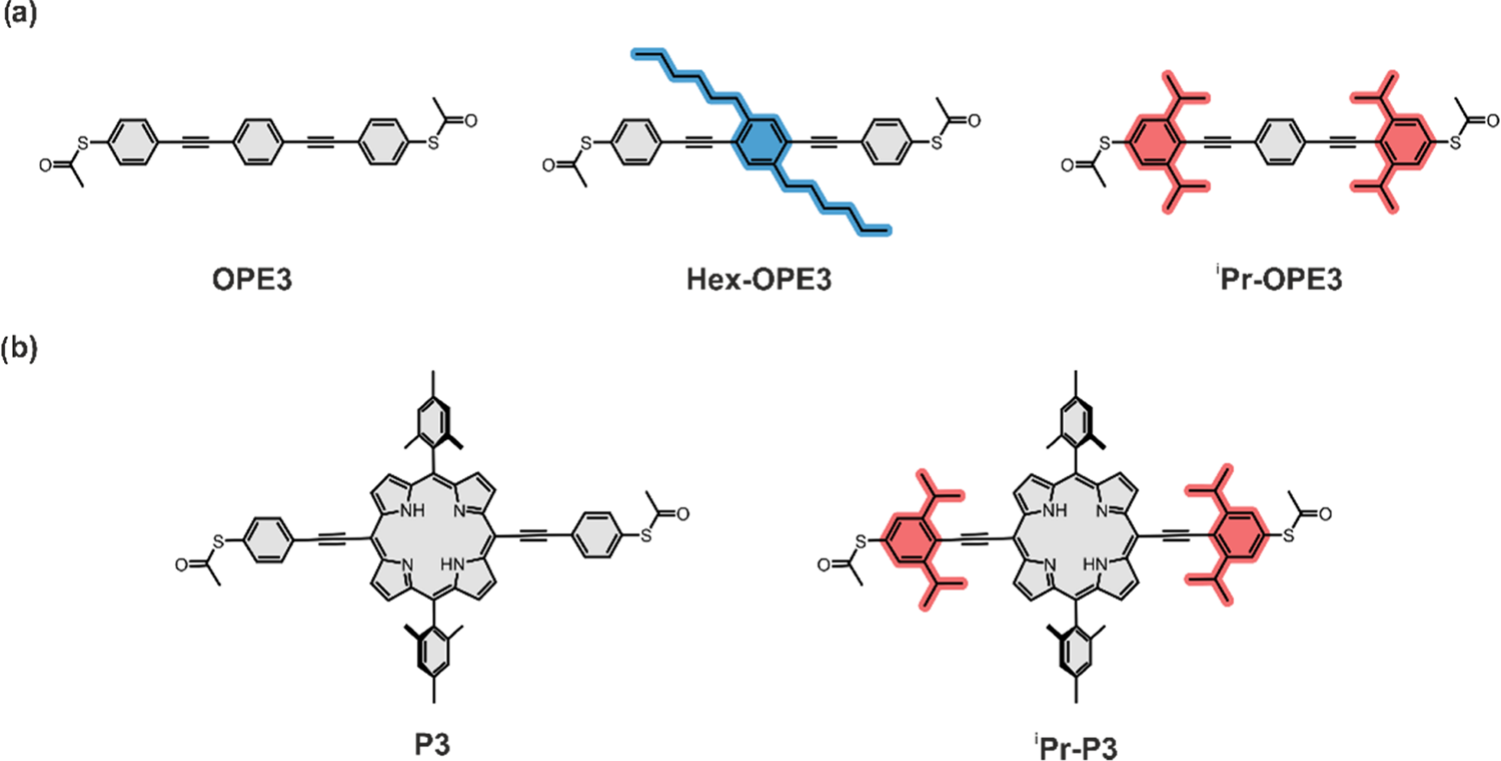
(a) Chemical structures of ****OPE3****, ****Hex-OPE3****, and ^**i**^**Pr-**OPE3****. Bulky group bearing phenyls are highlighted in blue (hexyl) and red (iso-propyl). (b) Chemical structures of ****P3**** and ^**i**^**Pr**-****P3****. Bulky groups bearing phenyls are highlighted in red (iso-propyl).

The chemical structures of the molecules included in this study can be found in Figure 1. The compounds ****OPE3**** (*S,S’*-((1,4-phenylenebis(ethyne-2,1-diyl))bis(4,1-phenylene))diethanethioate), ****Hex-OPE3**** (*S,S’*-(((2,5-dihexyl-1,4-phenylene)bis(ethyne-2,1-diyl))bis(4,1-phenylene))diethanethioate), and ^**i**^**Pr-**OPE3**** (*S,S’*-((1,4-phenylenebis(ethyne-2,1-diyl)bis(3,5-di-*iso*-propyl-4,1-phenylene))diethanethioate)) share the same backbone (Figure 1a), consisting of three linearly arranged phenyl units that are interlinked by acetylenes. The terminal phenyls are decorated with acetate-masked thiol groups, which undergo *in situ* deprotection on the gold surface of the MCBJ setup to establish a molecule-electrode contact. In the case of ****Hex-OPE3****, the central phenyl unit is decorated with two hexyl chains (as highlighted in blue in Figure 1a), whereas ^**i**^**Pr-**OPE3**** bears two *iso*-propyl chains on each of the anchoring group bearing terminal phenyls (as highlighted in red in Figure 1a). The compounds ****P3**** (*S,S’*-(((10,20-dimesitylporphyrin-5,15-diyl)bis(ethyne-2,1-diyl))bis(4,1-phenylene))diethanethioate) and ^**i**^**Pr-**P3**** (*S,S’*-(((10,20-dimesitylporphyrin-5,15-diyl)bis(ethyne-2,1-diyl))bis(3,5-di-*iso*-propyl-4,1-phenylene))diethanethioate) analogously share a similar linear backbone, but the central phenyl, in comparison to the OPE series, is substituted by a porphyrin with 2,4,6-trimethylbenzene (Mes, as abbreviation of its trivial names: Mesityl) units in the lateral position with respect to the anchoring group bearing phenyls (Figure 1b). In addition to the terminal anchor groups, ****P3**** bears lateral bulky mesitylene groups, thus mimicking ****Hex-OPE3**** in this class of compounds. The two *iso*-propyl chains on each of the anchoring group bearing phenyls of ^**i**^**Pr-**P3**** (highlighted in red in Figure 1b) make this compound the porphyrin analogue of ^**i**^**Pr-**OPE3**** in the OPE series.

The transport effect of decorating the molecules with the (molecules separating and electronically insulating) bulky groups (*iso*-propyl and hexyl) is investigated by the comparison of transport features of the individual classes of molecules (****OPE3**** with ****Hex-OPE3**and**^**i**^**Pr-**OPE3**** for the series of OPEs, and ****P3**** with **^**i**^Pr-P3** for the series of porphyrins) and further evaluated by cross-class comparison (****Hex-OPE3**** with ****P3**** and ^**i**^**Pr-**OPE3**** with ^**i**^**Pr-**P3****).

### Experimental – Synthesis

**Figure 2:**
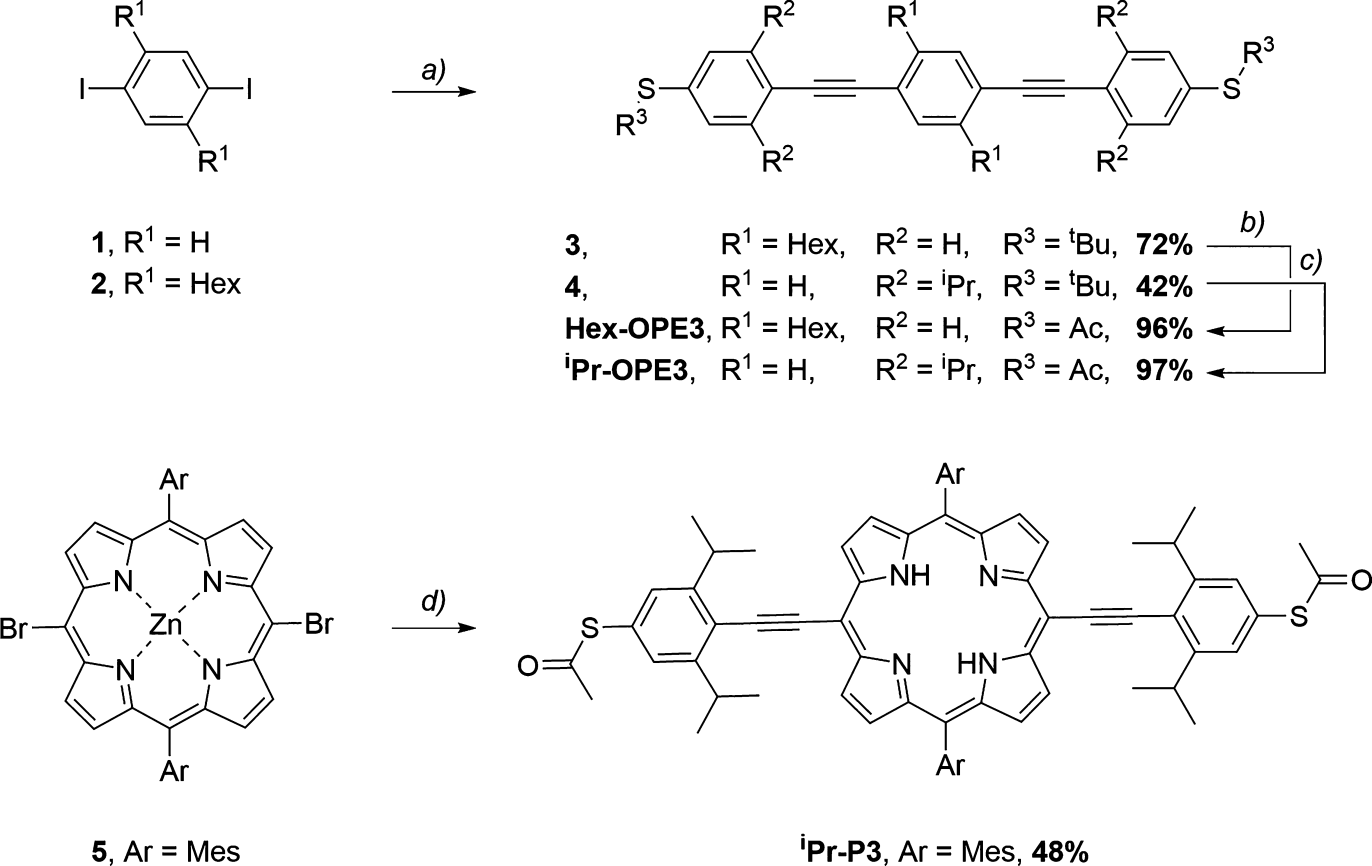
Synthetic overview. (a) Pd(PPh_3_)_2_Cl_2_, CuI, *tert*-butyl(4-ethynylphenyl)sulfane (for **3**) or *tert*-butyl(4-ethynyl-3,5-di-iso-propylphenyl)sulfane (for **4**), THF/NEt_3_ (1:1), 50 °C, 12 h. (b) Bi(OTf)_3_, AcCl, toluene/CH_3_CN (1:1), rt, 3 h. (c) Bi(OTf)_3_, AcCl, toluene/CH_3_CN (1:1), rt, 3 h. (d) (1) Pd(PPh_3_)_4_, CuI, *tert*-butyl(4-ethynyl-3,5-di-iso-propylphenyl)sulfane, toluene/NEt_3_ (4:1), 100 °C, 24 h; (2) Bi(OTf)_3_, AcCl, toluene/CH_3_CN (1:1), rt, 3 h.

Bare ****OPE3****(25) and ****P3****(10) were synthesized according to literature procedures. The bulky group bearing compounds ****Hex-OPE3****, ^**i**^**Pr-**OPE3****, and ^**i**^**Pr-**P3**** were synthesized in a two-step linear approach as shown in Figure 2. Starting from commercially available aryl-halide 1, and literature known **2**(26) and **5**,^27^ palladium-mediated Sonogashira–Hagihara cross-coupling with *tert*-butyl(4-ethynylphenyl)sulfane (in the reaction with **1**) or *tert*-butyl(4-ethynyl-3,5-di-iso-propylphenyl)sulfane^19^ (in the reactions with **2** and **5**) followed by bismuth(III)trifluoromethanesulfonate-mediated trans-protection^28^ from *tert*-butyl to acetate-masked thiophenols gave access to ****Hex-OPE3****, ^**i**^**Pr-**OPE3****, and ^**i**^**Pr-**P3****, respectively. The purity and identity of all compounds were fully corroborated by ^1^H-nuclear-magnetic resonance (NMR) and ^13^C{^1^H}-NMR spectroscopy as well as high-resolution electron spray-ionization mass spectrometry (HR-ESI-MS). ^**i**^**Pr-**P3**** was further characterized by UV–vis spectroscopy. Detailed experimental procedures and spectroscopic data can be found in Section S1.

### Experimental – Transport investigations

Samples consist of a thin gold constriction, which is suspended on an insulating layer of polyimide deposited on top of a flexible substrate. The sample is clamped between two lateral supports, mounted in a three-point bending mechanism with a central pushing rod that is operated by a piezoelectric actuator. Upon bending of the substrate, by pushing the central rod, the gold wire stretches. At some point, the gold wire breaks, leaving two atomically sharp electrodes whose separation can be adjusted mechanically with picometer resolution.^29^ The wire can be fused back by unbending the substrate, thereby decreasing the electrodes’ distance.

This breaking–making process can be repeated thousands of times while the conductance of the junction is recorded. The data shown in this manuscript are the two-dimensional (2D) histograms of the conductance plotted against the displacement, which is built from the individual “breaking traces”. The conductance *G* is plotted in units of *G*_0_, the quantum of conductance with *G*_0_ = 2*e*^2^/*h* (*e* is the elementary charge and *h* is the Plank constant, whereas 1 *G*_0_ is the conductance of a single gold atom bridging the junction, just before the wire breaks); the displacement is plotted in units of nanometers (nm). The measurements were performed in an in-house built MCBJ setup at room temperature in ambient conditions while applying a constant bias voltage of 100 mV across the two electrodes (more details are presented in the Supporting Information). Each sample was first characterized for 1000 consecutive breaking traces to assess its cleanliness. Then, about 5 μL of a solution containing the target molecule in CH_2_Cl_2_ was drop-cast on the device (5 μM for the **OPE3**s and 10 μM for the ^**i**^**Pr-**P3****) and the measurements were started. Detailed information about the MCBJ setup and the used methods were published earlier.^9,29,30^

## Results

**Figure 3:**
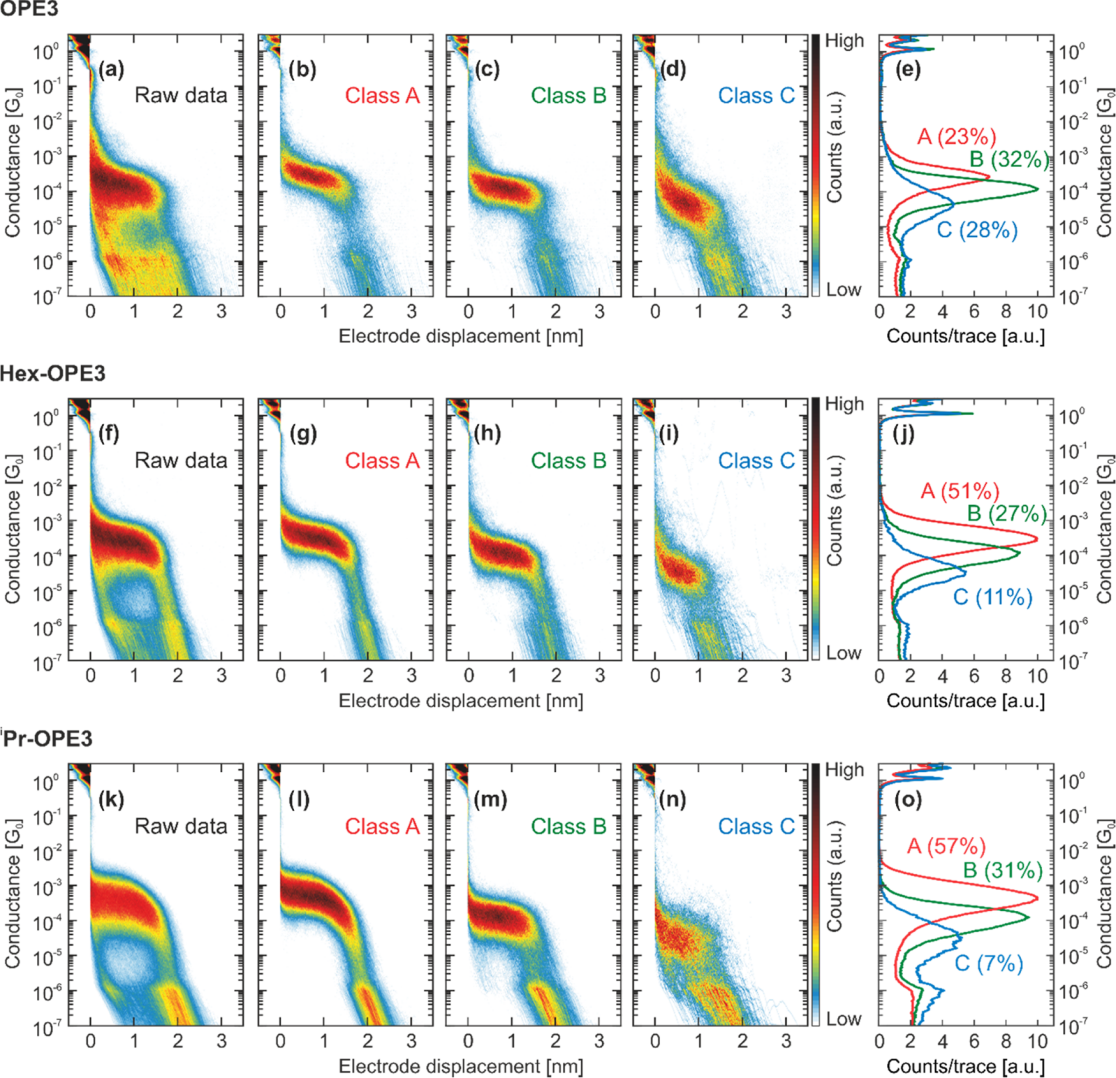
(a, f, k) Two-dimensional (2D) conductance vs displacement histograms of ****OPE3****, ********Hex-********, and **^i^Pr-OPE3**, respectively. No data selection was made. (b, g, l) 2D conductance vs displacement histogram of Class A breaking traces of **OPE3**, **Hex-OPE3**, and **^i^Pr-OPE3**, respectively, obtained by our reference-free cluster analysis. (c, h, m) 2D conductance vs displacement histogram of Class B breaking traces of **OPE3**, **Hex-OPE3**, and **^i^Pr-OPE3**, respectively, obtained by our reference-free cluster analysis. (d, i, n) 2D conductance vs displacement histogram of Class C breaking traces of **OPE3**, **Hex-OPE3**, and **^i^Pr-OPE3**, respectively, obtained by our reference-free cluster analysis. (e, j, o) One-dimensional (1D) conductance histograms corresponding to classes A, B, and C of **OPE3**, **Hex-OPE3**, and **^i^Pr-OPE3**, respectively. The percentages shown next to the 1D histograms are the corresponding yields of the class with respect to the number of molecular traces.

The two-dimensional (2D) conductance vs displacement and one-dimensional (1D) conductance histograms plotted from the breaking traces of the MCBJ experiments on the OPE series ****OPE3****, ****Hex-OPE3****, and ^**i**^**Pr-**OPE3**** can be found in Figure 3.

In all measurements comprising the raw data (left panels; Figure 3a,f,k for ****OPE3****, ****Hex-OPE3****, and ^**i**^**Pr-**OPE3****, respectively), a molecular conductance feature is present at a conductance of the order of 10^–4^*G*_0_ with a length of less than 2 nm. Throughout the measured molecules, these plateaus differ mainly in shape and relative intensity. While ****OPE3**** shows a rather flat and narrow plateau, the bulky group bearing analogues ****Hex-OPE3**** and ^**i**^**Pr-**OPE3**** show more slanted plateaus with a wider spread in conductance, pointing to the existence of more diverse configurations between the molecules and the electrodes compared to bare ****OPE3****. The 2D histogram of ****OPE3**** in Figure 3a presents more intense features at around 10^–6^*G*_0_, while in the same area in the histograms of ****Hex-OPE3**** and ^**i**^**Pr-**OPE3**** (Figure 3f,k), hardly any counts are visible.

To further investigate the transport phenomena, a reference-free unsupervised clustering algorithm^9^ was used to subdivide the different data sets into different classes. The approach uses a combination of the 1D and 2D histograms constructed from each breaking trace (more details can be found in Section S2). The *k-means++* algorithm is applied to partition the data set in *k* clusters, in which each element of the data set is assigned to the cluster with the closest mean. It consists of an iterative procedure that aims at finding the partition with the lowest within-cluster variance. This method is easily translatable across different molecules, only requiring the user to select the limits of the histograms and the number of clusters *k*.

In this study, we selected four classes as this allows to separate most of the molecular features while avoiding overclustering (i.e., splitting the same plateau in two different classes). Class A, B, and C are associated with molecular features and ordered after decreasing conductance (*G*(A) > *G*(B) > *G*(C)). Furthermore, we observe a class D (see Figures S17–S19 panels (a)) showing the exponential conductance decay typical of tunneling associated with empty junctions (i.e., without a molecule bridging the electrodes). The 2D histograms of each conductance class (A–C) for each member of the OPE series can be found in Figure 3 (b–d for ****OPE3****, g–i for ****Hex-OPE3****, l–n for ^**i**^**Pr-**OPE3****). The key findings for each molecule are listed in the following. Class A for ****OPE3**** shows a rather flat, slightly sloped conductance plateau with an averaged peak position at 2.6 × 10^–4^*G*_0_ and a full width at half-maximum (FWHM) of 0.8 decades. It is built from 23% of all molecular traces. Class B looks almost like class A but with a slightly straighter plateau at a lower conductance (at 1.2 × 10^–4^*G*_0_ and a FWHM of 0.8 decades, with a junction formation probability of 32% of all molecular traces). Class C shows a sloped and broad plateau with an average peak position at 4.3 × 10^–5^*G*_0_, a FWHM of 1.2 decades, and a yield of 28% of all molecular traces. The information obtained from the clustering of the ****OPE3**** molecules is summarized in Table 1. Notice that the yields of these classes do not add up to 100% because of an additional class that is discussed later.

**Table 1:** Summary of the Most Probable Conductance, Full Width at Half-Maximum, Length, Relative, and Total Yield Obtained for Classes A, B, and C of ****OPE3****, ****Hex-OPE3****, and ^**i**^**Pr-**OPE3****^a^

molecule	class	conductance (*G*_0_)	FWHM (decades)	length (nm)	relative yield (%)	total yield (%)
****OPE3****	A	2.6 × 10^–4^	0.8	1.4	23	14
	B	1.2 × 10^–4^	0.8	1.5	32	19
	C	4.3 × 10^–5^	1.2	1.2	28	17
****Hex-OPE3****	A	3.0 × 10^–4^	0.9	1.5	51	39
	B	1.1 × 10^–4^	0.9	1.4	27	21
	C	3.4 × 10^–5^	1.0	1.0	11	8
^**i**^**Pr-**OPE3****	A	4.3 × 10^–4^	1.1	1.5	57	48
	B	1.2 × 10^–4^	1.0	1.4	31	26
	C	2.8 × 10^–5^	1.5	1.1	7	6

a More information can be found in Table S1.

For ****Hex-OPE3**** (^**i**^**Pr-**OPE3****), class A appears as a slightly angled plateau that is slanting down just before rupture of the molecular junction at around 2 nm with an averaged peak position at 3.0 × 10^–4^*G*_0_ (4.3 × 10^–4^*G*_0_), a FWHM of 0.9 (1.1) decades, and a relative molecular yield of 51% (57%). Class B is very similar for the two molecules with an averaged peak position at 1.1 × 10^–4^*G*_0_ (1.2 × 10^–4^*G*_0_) and a FWHM of 0.9 (1.0) decades is similar to class B of ****OPE3****; the formation probability is 27% (31%). Class C also shows similarities across the molecules. For ****Hex-OPE3****, with a most probable conductance of 3.4 × 10^–5^*G*_0_ and a FWHM of 1.0 decades, it appears less pronounced but similarly shaped in comparison to the class C of ****OPE3****, with a relative molecular yield of 8%. Class C of ^**i**^**Pr-**OPE3****, with a relative molecular yield of 6%, shows a much broader plateau with a width of 1.5 decades but at a similar conductance value.

Although from the raw data of ****OPE3**** it is clear that there is a plateau at around 10^–6^*G*_0_, it is not easily isolated by clustering with the chosen parameters, and it is incorporated with empty traces in class D. Further clustering can be used to separate it (see Figures S17–S19 panels (b)), revealing a class at 1.7 × 10^–6^*G*_0_ with 10% yield and a length of about 1.4 nm. The same analysis presents a similar subclass for ****Hex-OPE3****, with a 1.3 nm long plateau at 1.3 × 10^–6^*G*_0_, with a lower yield of 8%. However, in this case, the plateau is more slanted and less well-defined (see Figure S18b). Also, ^**i**^**Pr-**OPE3**** shows traces at around the same conductance value (1.3 × 10^–6^*G*_0_), with a similar length (1.6 nm) and yield (4%).

In summary, over the series of OPE′s (****OPE3****, ****Hex-OPE3****, and ^**i**^**Pr-**OPE3****), three trends can be observed: (i) Class A is dominant in ****Hex-OPE3**** and ^**i**^**Pr-**OPE3**** showing an angled conductance decay before rupture of the molecular junction for ****Hex-OPE3**** and more prominently for ^**i**^**Pr-**OPE3****. (ii) Class B is very similar for all three compounds, showing almost no dispersion in the most probable conduction value, and (iii) the largest changes are present for the low-conductance Class C.

**Figure 4:**
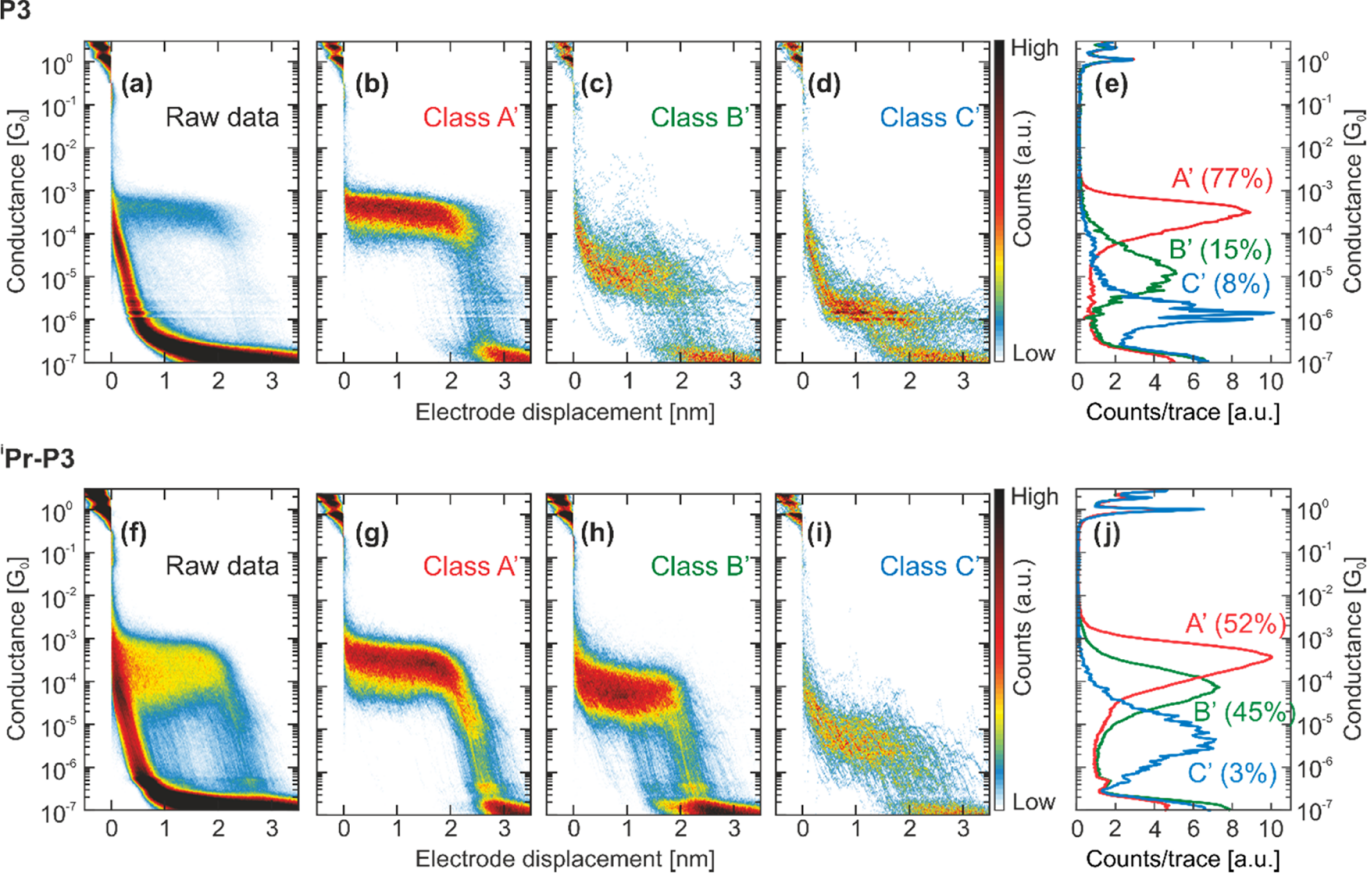
(a, f) 2D conductance vs displacement histograms of ****P3**** and ^**i**^**Pr**-****P3****, respectively. No data selection was made. (b, g) 2D conductance vs displacement histogram of Class A′ breaking traces of ****P3**** and ^**i**^**Pr**-****P3****, respectively, obtained by our reference-free cluster analysis. (c, h) 2D conductance vs displacement histogram of Class B′ breaking traces of ****P3**** and ^**i**^**Pr**-****P3****, respectively, obtained by our reference-free cluster analysis. (d, i) 2D conductance vs displacement histogram of Class C′ breaking traces of ****P3**** and ^**i**^**Pr**-****P3****, respectively, obtained by our reference-free cluster analysis. (e, j) 1D conductance histograms corresponding to classes A′, B′, and C′ of ****P3**** and ^**i**^**Pr-**P3****, respectively. The percentages shown next to the 1D histograms are the corresponding yields of the class with respect to the number of molecular traces.

For the porphyrins ****P3**** and ^**i**^**Pr-**P3****, the 2D conductance vs displacement and 1D conductance histograms plotted from the breaking traces are displayed in Figure 4. A clear molecular feature around a conductance of ∼5 × 10^–4^*G*_0_ reaching out to almost 3 nm is already present in the 2D histograms of the unfiltered breaking traces (Figure 4a,f for ****P3**** and ^**i**^**Pr-**P3****, respectively; the data of ****P3**** was already recorded in our previous study^15^). A distinct difference between the two histograms and the raw data is that the molecular feature in the case of ****P3**** is much better defined. Analogously to the OPE series, we have used the same unsupervised clustering procedure to separate the molecular features into four classes. The 2D histograms of each molecular conductance class (A′–C′) for each porphyrin can be found in Figure 4 (b–d for ****P3**** and g–i for ^**i**^**Pr–**P3****) and are discussed in the following. Class D′ comprises empty junctions, and the corresponding 2D histograms can be found in Figures S19–S21. Note that, although we label the classes A–D for the oligophenylethynylene series and A′–D′ for the two porphyrins, the physical origin of these classes may vary. Thus, a one-to-one correspondence of the classes between the two series of compounds is unlikely.

For ****P3****, Class A′ appears as a very flat and narrow plateau with a molecular yield of 77%, a peak position at 3.0 × 10^–4^*G*_0_, and a FWHM of 0.8 decades. Class B′, a slanted, broad cloud of conductance traces, is less frequently observed than Class A′, with a molecular yield of 15%, a peak position at 1.3 × 10^–5^*G*_0_, and a FWHM of 1.3 decades. Class C′ contains a narrow and flat plateau at low conductance with a most probable conductance of 1.4 × 10^–6^*G*_0_ and a FWHM of 0.7 decades. It is the least frequently observed class, with a relative yield of 8% of all breaking traces with molecular features.

Class A′ of ^**i**^**Pr-**P3**** displays an angled plateau that is slanting down just before rupture of the molecular junction at around 2 nm. The relative molecular junction formation probability is lower than for ****P3**** (52%) and the FWHM is 1.1 decades with a similar averaged peak position of 3.3 × 10^–**4**^*G*_0_, as compared to Class A’ of ****P3****. Compared to ****P3****, Class B′ has a higher conductance peak position at 7.1 × 10^–**5**^*G*_0_ and a FWHM of 1.1 decades but is much more frequently observed with a relative molecular yield of 45%; it thus appears as a well-defined plateau in the 2D histogram (Figure 4h). Class C′ displays a broad cloud of conductance traces, with a low relative molecular yield of 3%, an averaged conductance peak position at 4.6 × 10^–**6**^*G*_0_, and a FWHM of 1.5 decades. In summary, over the series of porphyrins (****P3**** and ^**i**^**Pr-**P3****), we observe that an angled conductance decay before rupture of the molecular junction appears in Class A′ for ^**i**^**Pr-**P3**** but not for ****P3**** and that Class B′ is much more frequently present in ^**i**^**Pr-**P3****. The clustering results for ****P3**** and ^**i**^**Pr-**P3**** are summarized in Table 2.

**Table 2:** Summary of the Most Probable Conductance, Full Width at Half-Maximum, Length, Relative, and Total Yield Obtained for Classes A′, B′, and C′ of ****P3****, ****Hex-OPE3****, and ****iPr-P3****^a^

molecule	class	conductance (*G*_0_)	FWHM (decades)	length (nm)	relative yield (%)	total yield (%)
****P3****	A′	3.0 × 10^–4^	0.8	2.5	77	10
	B′	1.3 × 10^–5^	1.3	1.9	15	2
	C′	1.4 × 10^–6^	0.7	1.1	8	1
^**i**^**Pr**-****P3****	A′	3.0 × 10^–4^	3.3 × 10^–4^	1.1	2.1	43
	B′	1.1 × 10^–4^	7.1 × 10^–5^	1.1	2.5	38
	C′	3.4 × 10^–5^	4.6 × 10^–6^	1.5	1.8	3

a More information can be found in Table S1.

## Discussion

First, we note that all three ****OPE3**** derivatives (****OPE3****, ****Hex-OPE3****, and ^**i**^**Pr-**OPE3****) display comparable conductance features. A detailed inspection is required to identify the effects of the decorating alkyl groups. This is expected since the three compounds have the same molecular backbones and corroborate the claim of forming single-molecule junctions. We thus hypothesize that the three very comparable classes observed for all three model compounds have the same physical chemical origin. The presence of multiple classes resides in the stochastic nature of the break-junction experiments: After every junction rupture, the electrodes are fully brought together to form a new connection. As such, the molecules can rearrange on the surface, yielding different local configurations and/or binding to sites on the gold surface with different coordinations. The possible origin of the observed classes is discussed in the following.

**Figure 5:**
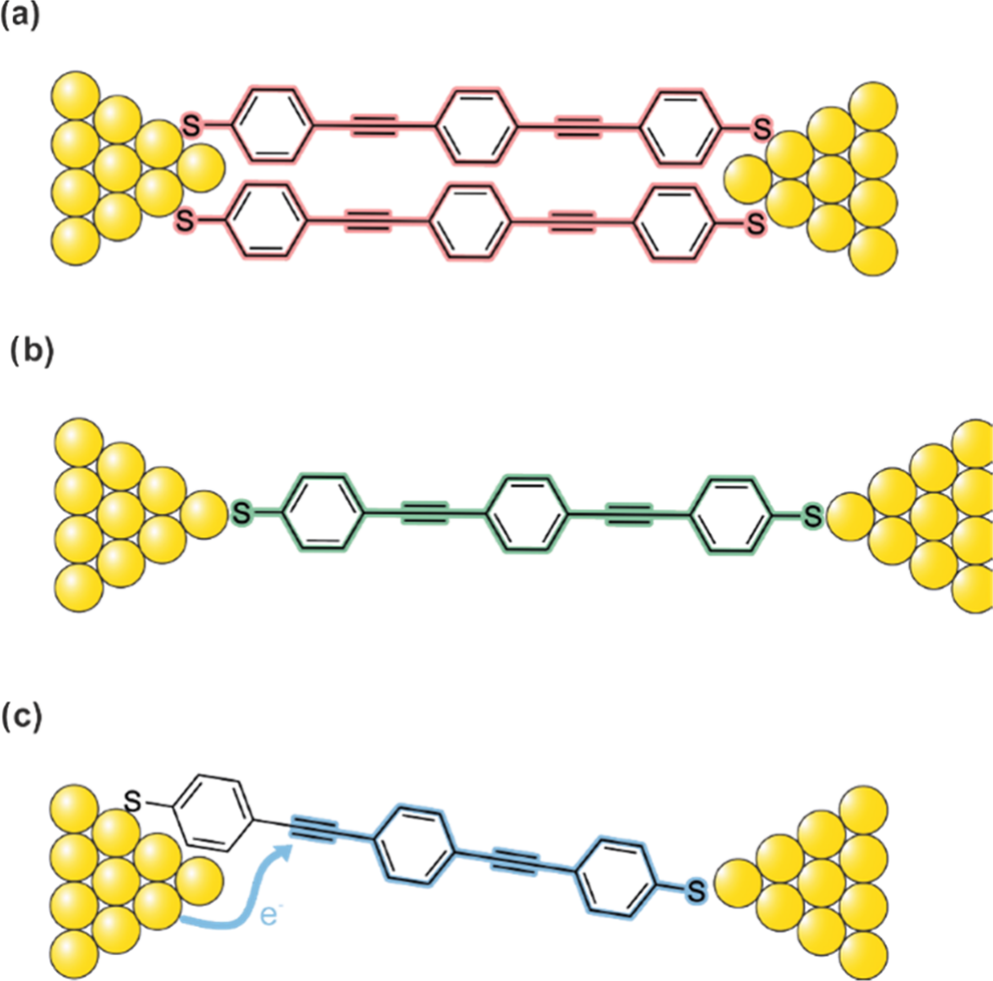
Schematic representation of possible interpretations of (a) Class A, (b) Class B, and (c) Class C for ****OPE3****.

Class A and B of ****OPE3**** are compatible with classes of ****OPE3**** reported by Cabosart et al.^9^ In their study, they observed a class with a conductance of 1 × 10^–4^*G*_0_, which is systematically found in all ****OPE3**** measurements with the same conductance and length. Thus, they assign this class to the junction in the single-molecule configuration (Figure 5b), in which the molecule is connected to the electrodes with the terminal sulfur atom on both sides. In our study, we observe the exact same behavior for Class B: the conductance values and lengths match the ones of previous studies, and they are the same across the three compounds. This reinforces the hypothesis of electronically insulating side groups since the class assigned to single-molecule conductance does not show differences when these are introduced.

Cabosart et al.^9^ also observed the presence of a group of traces at higher conductance than the single-molecule class, whose presence was more relevant in measurements with high yield. However, its origin is not completely clear. They proposed that it consists of junctions with more than one molecule in parallel (Figure 5a) or molecules for which injection occurs through space along the backbone (Figure 5c). Class A of this study presents remarkable similarities to this behavior, having larger conductance than Class B and presenting significant differences among ****OPE3****, ****Hex-OPE3****, and ^**i**^**Pr-**OPE3****.

As for Class C, its origin has not been thoroughly described in literature before, although we note that similar features have been reported for ****OPE3**** derivatives.^31^ One possible interpretation is that this corresponds to junction configurations where electrons are injected from the Au to the π-system of the molecule via phenyls or acetylenes (Figure 5c).

The trends across the classes in the family of the OPEs are consistent with the proposed explanations. Class B is identical across the three compounds: as the “bulky” substituents are inert from the electronic point of view, their effect on the main conduction path is negligible. Class C is also very similar across the three molecules, which indicates that the shielding effect of the bulky groups on the different injection paths is small. As expected from steric hindrance, the yield of Class C relative to the number of molecular traces decreases for ****Hex-OPE3**** and ^**i**^**Pr-**OPE3****. However, this interpretation needs to be handled with care as the yield of different classes can vary significantly from measurement to measurement,^9^ and it is at best a qualitative measure for changes in the behavior of the molecules.

Class A displays peculiar behavior. First, it presents significant increases in conductance from ****OPE3**** to ****Hex-OPE3**** and ^**i**^**Pr-**OPE3****, the latter having the highest value. Second, its shape changes significantly: For ****Hex-OPE3****, a faint tilt is present at the end of the plateau, just before rupture, which becomes very pronounced in ^**i**^**Pr-**OPE3****. The tilting indicates that the molecular junction changes significantly during the stretching process.

This change could be induced by conformational changes within the molecule over the stretching process.^32−35^ However, the use of rigid planar molecules and the absence of a typically observed change in conductance between two stable values, rather than a slope, making this explanation less likely.

Alternatively, the slope could be caused by an evolution of different electrode-molecule configurations over the course of the breaking process. We propose that the presence of alkyl substituents allows the observation of additional junction configurations by mechanically stabilizing those mechanically. This stabilization can occur by molecule-electrode or by molecule–molecule interactions with more than one molecule present in the junction. The fact that the titling occurs at the end of the plateau at the same position as for ****OPE3**** (when it is fully stretched) seems to favor our hypothesis. Interestingly, the tail appears to terminate at conductance values close to the ones of Class B, suggesting that the stretching process in traces belonging to Class A terminates with a similar geometry to Class B, i.e., the fully stretched sulfur-to-sulfur configuration.

Differences in the bonding coordination to the gold surface can also lead to changes in conductance even of an order of magnitude.^36−39^ However, this explanation is not compatible with the observations in this study. In fact, we observe changes in Class A with the addition of alkyl groups but not in Class B. This suggests that another factor is at the origin of the difference between these classes. For classes B and C, we do not observe changes across our series of molecules, and as such, we cannot exclude the difference in anchoring site as an explanation of the different conductance.

We also observe conductance traces at around 10^–6^*G*_0_, which may be related to π-stacking.^12,13^ However, such traces are present in all three molecules with similar conductances and comparable yield. The two possible explanations are that (i) the addition of bulky groups does not significantly hinder the formation of π-stacks when the molecules are deposited on the surface, or (ii) the origin of this plateau is not π-stacking. Since the length of the plateaus is close to that of Class B for all the molecules, the latter explanation seems more likely. The origin of the plateau may be related to different binding coordination to the gold surface.^36,39−41^

The trends discussed for the OPE molecules are also observed in porphyrin series ****P3**** and ^**i**^**Pr-**P3****. First, Class A’ has a very similar most probable conductance value for the two (note that ****P3**** is more similar to ****Hex-OPE3**** than to ****OPE3**** since ****P3**** is bearing six insulating methyl groups on the phenyls laterally connected to the porphyrin already). However, we again observe the development of a pronounced tilted area at the end of the plateau for the compound, with *iso*-propyl groups close to the anchoring sulfur atoms. Differently from ****OPE3****, in ****P3**** we do not observe two distinct high-conductance classes. There are two possible hypotheses: first, that the mechanism that gives rise to Class A for ****OPE3**** is not present for ****P3****; second, that they are so similar in ****P3**** that our analysis cannot distinguish between them. The first hypothesis seems unlikely since the mechanical stabilization mechanism we proposed for ****OPE3**** should also play a role for ****P3****. The second hypothesis, on the other hand, is supported by Class B’ of ^**i**^**Pr-**P3****, which is more similar to Class A′ than to Class B’ of ****P3****. This indicates that in the case of ^**i**^**Pr-**P3****, we have two high-conductance classes, while in ****P3**** we do not observe them, most likely because they are too similar. Additionally, notice that Class B′ of ^**i**^**Pr-**P3**** has a shorter length than that of Class A’. This observation hints at a conduction pathway that is shorter than that defined by the sulfur-to-sulfur distance, albeit less efficient. It could be that the alkyl side groups mechanically stabilize pathways involving direct through-space injection into the π-system of the molecule. The other main difference between ****P3**** and of ^**i**^**Pr-**P3**** is that in the latter, we cannot isolate features similar to C′ of the former. However, we avoid deriving conclusions from this fact since the yield of C’ in ****P3**** is very low to begin with.

As the tilt of traces before rupture occurs for both families of compounds, one can assume that the behavior originates from the presence of alkyl spacers rather than from the molecular backbone itself. An explanation can be the corroding effect that thiolates can have on gold surfaces.^20,42−44^ As the junction is stretched, gold atoms can be pulled along the gold surface and eventually pulled out of it. This effect can be intensified in the presence of additional alkyl groups added to the molecular backbone: The presence of additional weak electrostatic interactions (London dispersion forces) can stabilize the formation of junctions where two (or more) molecules are bridging the gap and connected on both sides. This configuration is different from the stacked one often analyzed in literature^12,13,45^ in that (i) both molecules are connected on both sides and (ii) it presents one “locked” configuration, rather than two molecules that can slide on each other to form dimers of different total length.

**Figure 6:**
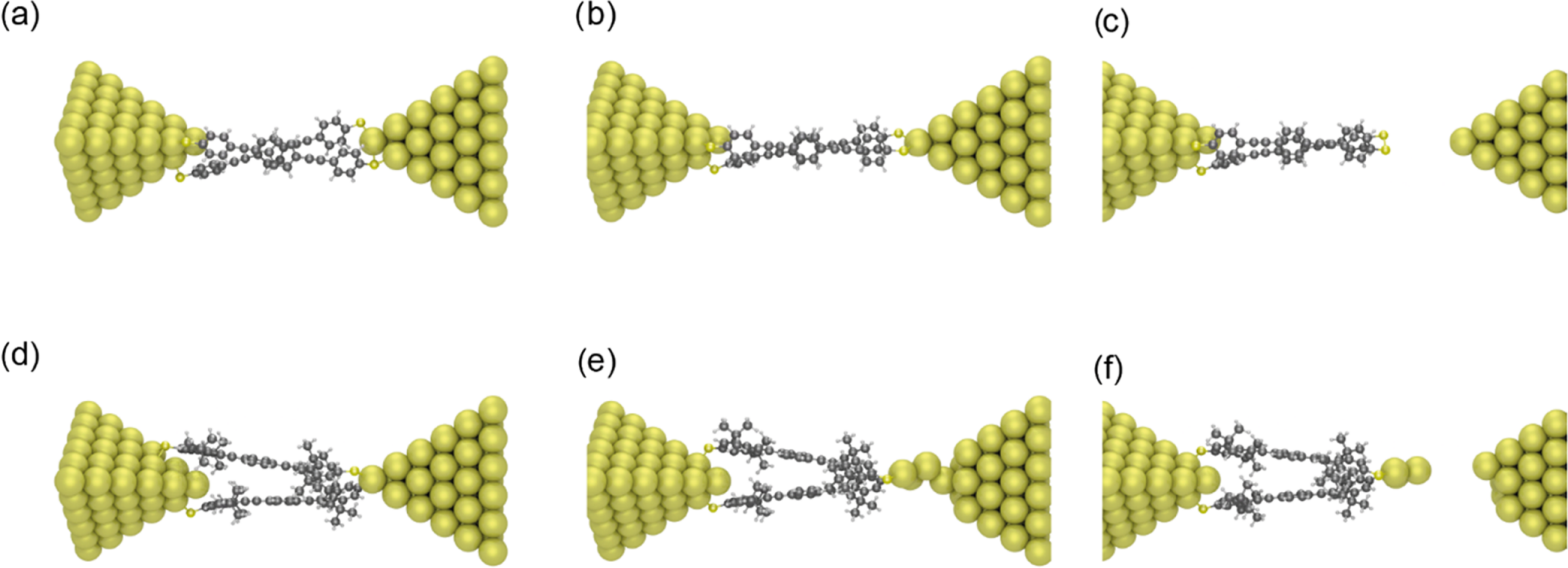
Snapshots of the pulling process obtained by DFT for (a–c) ****OPE3**** and for (d–f) **^i^Pr-**OPE3****.

To better illustrate this concept, we performed density functional theory (DFT) calculations that simulate the pulling process for the three ****OPE3**** compounds used in this study. The calculations were performed with the ADF package,^46,47^ similar to previous studies that performed comparable calculations,^30,48^ using the GGA-PBE functional,^49^ and the triple-ζ plus polarization (TZP) basis set. The zeroth order regular approximation (ZORA) to the Dirac equation was used to account for relativistic effects in the electrodes. Each molecule is connected to two pyramidal gold electrodes. The geometry was converged to energy changes of less than 10^–3^ hartree, energy gradients of less than 10^–3^ hartree/Å maximum and 6.7 × 10^–4^ hartree/Å RMS while keeping the position of the outer layer of gold atoms for each electrode fixed. We then separated the gold electrodes in steps of 4 pm (while again keeping the outer layers fixed), and for each new gap size, we relaxed the geometry of the molecular junction. Some snapshots resulting from this process are displayed in Figure 6.

Let us first consider the standard situation, starting with Figure 6a, in which an open junction contains two ****OPE3**** molecules. If the junction is stretched further, reaching the full length of the ****OPE3**** molecule (Figure 6b), there is no change in the electrode surface: the molecules have just slid on the surface. Eventually, if the junction is stretched beyond the rupture point (Figure 6c), then the molecules simply disconnect from one of the electrodes.

A different rupture process could occur, as illustrated in Figure 6d–f. In Figure 6d, the junction is in a configuration similar to that in Figure 6a, although here we used two ^**i**^**Pr-**OPE3**** molecules. When the junction opens more, the molecules start dragging gold atoms off the surface (Figure 6e). Eventually, they are completely pulled out of the surface (Figure 6d), leading to the rupture of the junction. A proper statistical analysis of the simulation of the rupture of the junction over several junctions would be needed to assess if the latter mechanism is a result of the addition of bulky alkyl groups to the backbone. However, given the very high computational cost for each of such simulated breaking traces, it is unfeasible to perform such calculations in the same large statistics as those done experimentally. Nevertheless, this illustrates our current working hypothesis of the formation of the tail at the end of the conductance plateaus in the measurements of the substituted OPEs and porphyrins.

## Conclusions

We have shown that the nature, number, and position of alkyl substituents on aromatic compounds play an important role in the transport features of single-molecule junction experiments, even though they are expected to be insulating moieties and used as substituents to minimize molecular interactions. We propose that the groups can mechanically stabilize different molecule–molecule and molecule-electrode configurations by weak electrostatic interactions (London dispersion forces). The latter seems to be especially prominent when the alkyl substituents are positioned in the proximity of the anchoring groups. Furthermore, the addition of bulky side groups does not significantly influence the presence of conductance traces at 10^–6^*G*_0_ in OPEs. Thus, the placement, number, and length of alkyl chains in aromatic compounds for molecular electronics must be carefully designed depending on the purpose of the individual experiment, balancing the effects of increased solubility and the impact they have on the variety of junction configurations.

## Data Availability

The MCBJ raw data is available free of charge at: https://doi.org/10.4121/d4cbdc52-b7e3-4fee-b00d-a29cd47dbf48.

## Supporting Information Available

Remarks, procedures, and analytical data (H NMR, HMRS, UV/vis) of the synthesis of the compounds in this study; MCBJ measurement and clustering details, clustering results, additional two-dimensional conductance-displacement histograms, and summary table with analysis results (PDF)
