# Supplementary material for: Influence of Peripheral Alkyl Groups on Junction Configurations in Single-Molecule Electronics
Source: J Phys Chem C Nanomater Interfaces. 2024 Jan 16;128(3):1413–22. doi: 10.1021/acs.jpcc.3c06970 (PMC10823531; doi:10.1021/acs.jpcc.3c06970)
Supplement: Supplementary file 1 — jp3c06970_si_001.pdf [file jp3c06970_si_001.pdf]

Supporting Information

Influence of Peripheral Alkyl Groups on Junction Configurations in Single-Molecule Electronics

Luca Ornago,[a#] Patrick Zwick,[b#] Sebastiaan van der Poel,[a] Thomas Brandl,[b] Maria El Abbassi,[a] Mickael L. Perrin,[c,d,e] Diana Dulić,[f] Herre S. J. van der Zant*,[a] and Marcel Mayor*,[b, g, h]*

[a] Kavli Institute of Nanoscience, Delft University of Technology, Lorentzweg 1, 2628 CJ Delft, The Netherlands

[b] Department of Chemistry, University of Basel, St. Johanns-Ring 19, 4056 Basel, Switzerland

[c] Transport at Nanoscale Interfaces Laboratory, Empa, Swiss Federal Laboratories for Materials Science and Technology, 8600 Dübendorf, Switzerland

[d] Department of Information Technology and Electrical Engineering, ETH Zürich, 8092 Zürich, Switzerland

[e] Quantum Center, ETH Zürich, 8093 Zürich, Switzerland

[f] Department of Physics and Department of Electrical Engineering, Faculty of Physical and Mathematical Sciences, University of Chile, Avenida Blanco Encalada 2008, Santiago, 8330015, Chile.

[g] Institute for Nanotechnology (INT), Karlsruhe Institute of Technology (KIT), P.O. Box 3640, 76021 Karlsruhe, Germany

[h] Lehn Institute of Functional Materials (LIFM), School of Chemistry, Sun Yat-Sen University (SYSU), 510275 Guangzhou, China

[#] These authors made equal contributions

Table of Contents

S1 Synthesis 3
S1.1 General Remarks 3
S1.2 Experimental Procedures and Analytical Data 4
S2 Mechanically Controllable Break Junction (MCBJ) Measurements 20
S2.1 Clustering Parameters 20
S2.2 Supplementary Plots and Tables 21
S3 References 23

S1 Synthesis

S1.1 General Remarks

All commercially available chemicals were used without further purification. NMR solvents were obtained from CIL Cambridge Isotope Laboratories, Inc. (Andover, MA, USA) or Sigma-Aldrich. All NMR experiments were performed on Bruker Avance III or III HD, two or four-channel NMR spectrometer operating at 400.13 or 500.13 MHz proton frequency. The instruments were equipped with direct observe BBFO, indirect BBI or cryogenic four-channel QCI (H/C/N/F) 5 mm probes all with self-shielded z-gradient. The experiments were performed at 298K or 295K. All chemical shifts (δ) are reported in parts per million (ppm) relative to the used solvent and coupling constants (J) are given in Hertz (Hz). The multiplicities are written as: s = singlet, d = doublet, t = triplet, q = quartet, hept = heptet, m = multiplet. Flash column chromatography (FCC) was performed with SiliaFlash® P60 from SILICYCLE with a particle size of 40-63 µm (230-400 mesh) and for TLC Silica gel 60 F_{254} glass plates with a thickness of 0.25 mm from Merck were used. The detection was observed with a UV-lamp at 254 or 366 nm. Gel Permeation Chromatography (GPC) was performed on a Shimadzu Prominence System with PSS SDV preparative columns from PSS (2 columns in series: 600 mm x 20.0 mm, 5 µm particles, linear porosity “S“, operating ranges: 100 – 100 000 g.mol-1) using chloroform as solvent. MALDI-TOF mass spectra were recorded on a Bruker MicroFlex LRF spectrometer using trans-2-[3-(4-tert-Butylphenyl)-2-methyl-propenylidene]malononitrile (DCTB) as a matrix. High resolution mass spectra (HRMS) were measured on a Bruker solariX – MALDI-FTICR-MSn.

S1.2 Experimental Procedures and Analytical Data

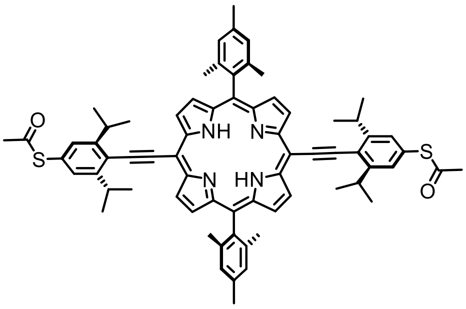

1

^{i}Pr-P3: A mixture of [5,15-dibromo-10,20-dimesitylporphyrinato]zinc(II) (30 mg, 39.3 µmol, 1.0 eq.) and *tert*-butyl(4-ethynyl-3,5-diisopropylphenyl)sulfane (23 mg, 86.5 µmol, 2.2 eq.), in toluene (20 mL) and NEt_3 (5 mL) in a 50 mL *Schlenk*-tube was degassed by purging with argon for 20 min. $Pd(PPh_3)_4$ (2.27 mg, 1.97 µmol, 0.05 eq.) and CuI (449 µg, 2.36 µmol, 0.06 eq.) were added and the tube was sealed and placed in a pre-heated oil bath at 100 °C for 1 d. The mixture was concentrated under reduced pressure and the residue, re-dissolved in CH_2Cl_2, was filtered over a silica plug eluted with CH_2Cl_2. The crude mixture was dried under vacuum and re-dissolved in dry and degassed toluene (5 mL) and MeCN (5 mL). Acetyl chloride (0.56 mL, 7.86 mmol, 200 eq.) and bismuth(III)trifluoromethanesulfonate (78.9 mg, 118 µmol, 3.0 eq.) were added, subsequently, and the mixture was stirred for 3 h until TLC monitoring indicated completion of the reaction. Toluene and water were added, and the organic phase was separated, dried over anhydrous Na_2SO_4, filtered over silica eluted by CH_2Cl_2, and concentrated under reduced pressure. The residue was subjected to flash column chromatography (SiO_2, *n*-heptane/CH_2Cl_2 (1:2)) followed by recrystallization from CH_2Cl_2/CH_3OH. The product **^{i}Pr-P3** (20 mg, 19 µmol, 48%) was isolated as a purple crystalline solid.

^{1}H-NMR (250 MHz, $CDCl_3$, 298 K, *δ*/ppm): 9.59 (d, $^{3}J_{HH}$ = 4.8 Hz, 4H), 8.72 (d, $^{3}J_{HH}$ = 4.7 Hz, 4H), 7.40 (s, 4H), 7.31 (s, 4H), 4.28 (p, $^{3}J_{HH}$ = 6.9 Hz, 4H), 2.65 (s, 6H), 2.51 (s, 6H), 1.89 (s, 12H), 1.60 (s, 12H), 1.58 (s, 12H), -1.73 (s, 2H).

^{13}C{^{1}H}-NMR (151 MHz, $CDCl_3$, 298 K, *δ*/ppm): 193.90, 152.16, 139.22, 138.08, 137.73, 128.60, 128.57, 127.90, 122.96, 120.43, 101.02, 100.86, 93.81, 32.53, 30.38, 23.72, 21.59, 21.46.

UV/VIS (CH_2Cl_2): λ_{max} [nm] = 444, 603, 693.

HRMS (ESI, -): *m/z* calcd. for $C_{70}H_{71}N_4O_2S_2$ $[M+H]^+$: 1063.5013, found: 1063.4993.

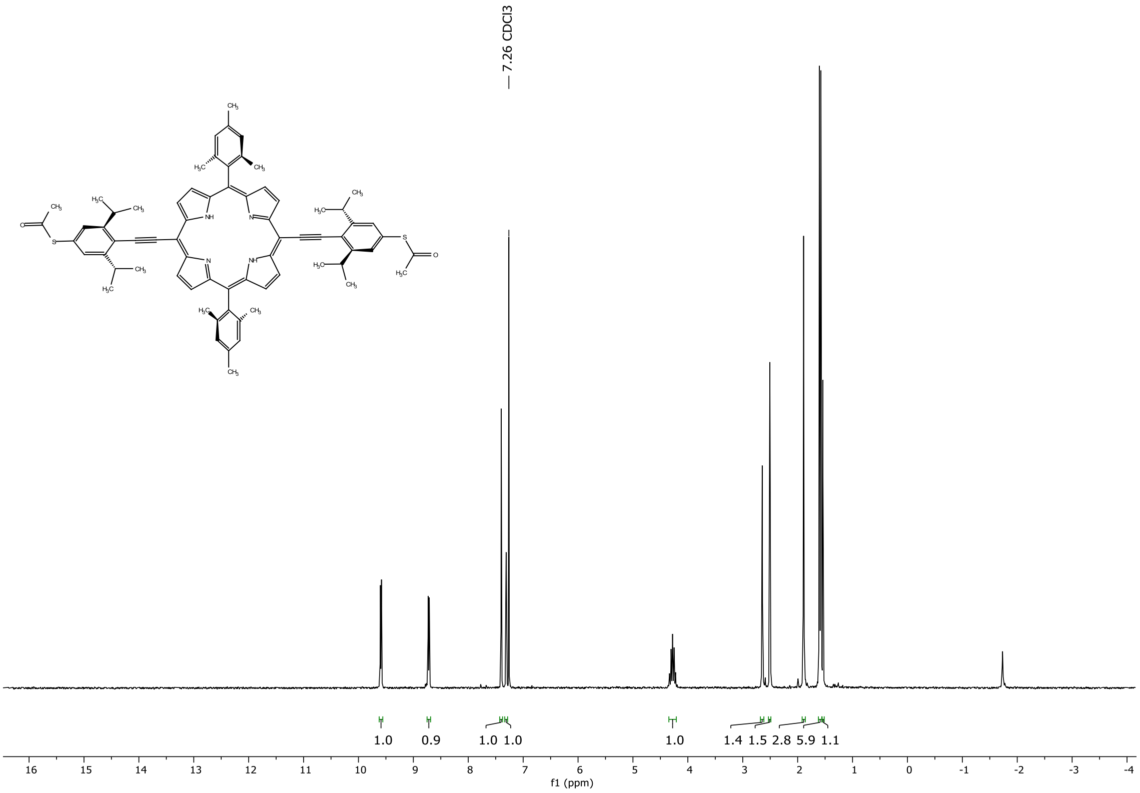

Figure S 1: **^{1}H-NMR** spectrum of **^{i}Pr-P3** in $CDCl_3$.

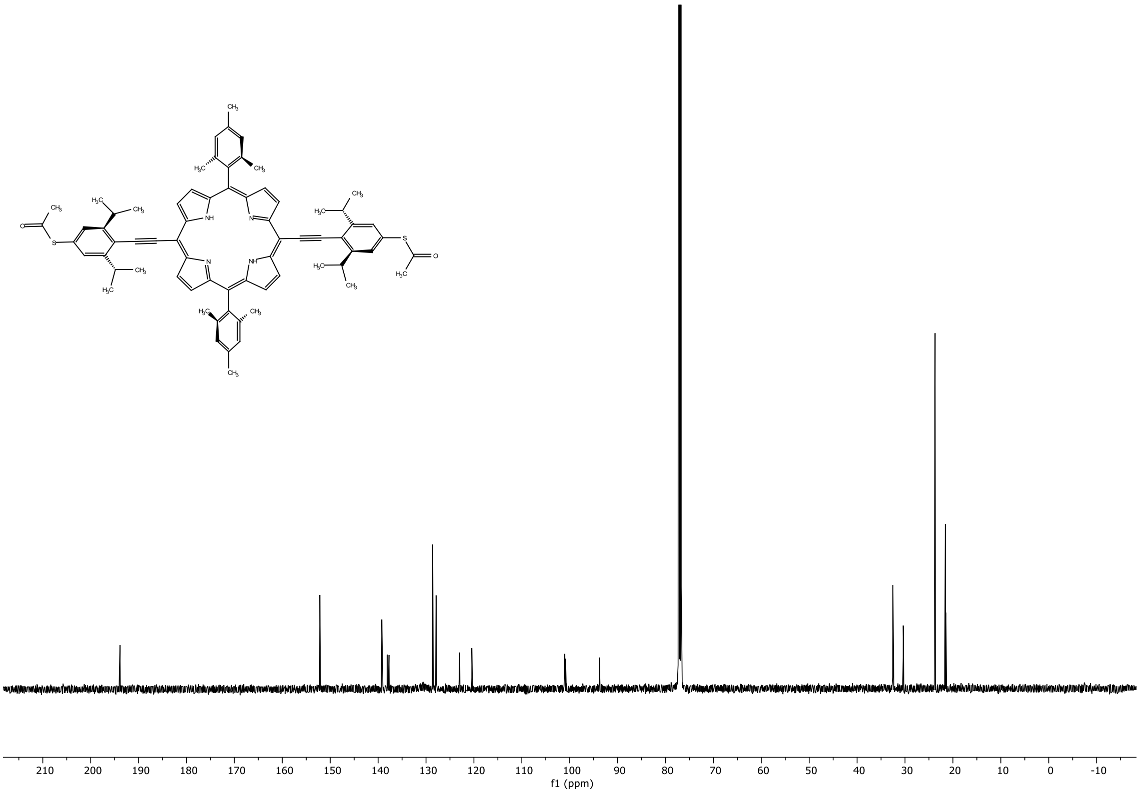

Figure S 2: **^{13}C{^{1}H}-NMR** spectrum of **^{i}Pr-P3** in $CDCl_3$.

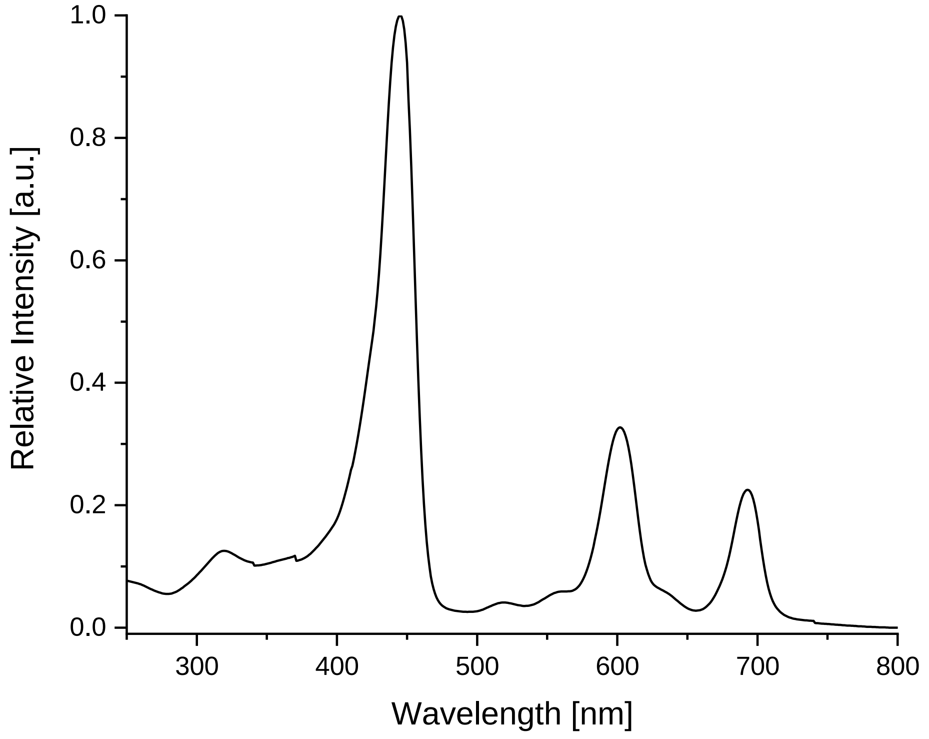

Figure S 3: **Normalized UV-vis** spectrum of ^{i}**Pr-P3** recorded in CH_2Cl_2.

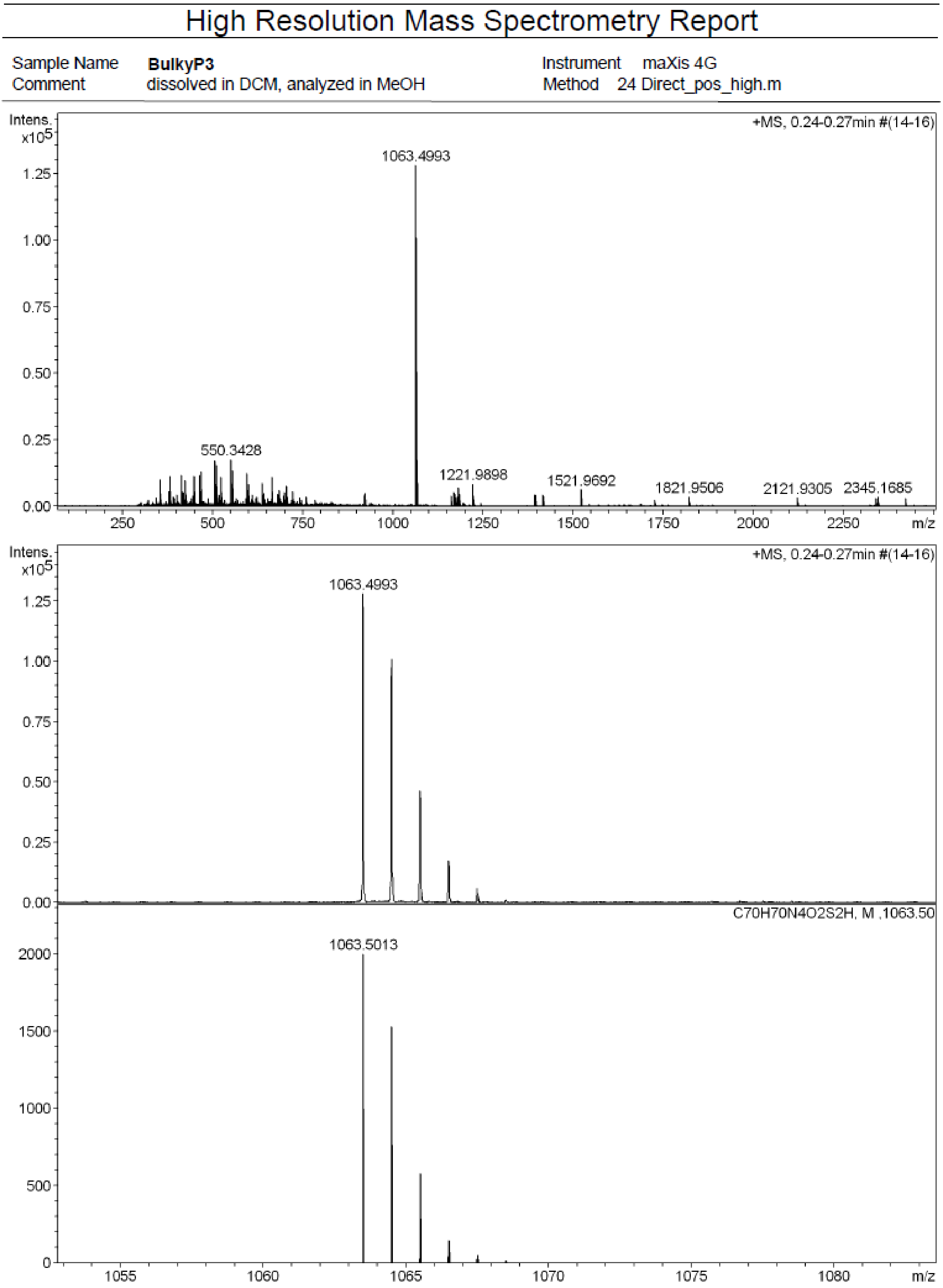

Figure S 4: **HRMS (ESI, +)** spectrum of **^{i}Pr-P3.**

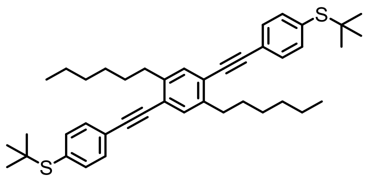

(((2,5-dihexyl-1,4-phenylene)bis(ethyne-2,1-diyl))bis(4,1-phenylene))bis(*tert*-butylsulfane) (3)

A 100 mL round bottom flask was purged with argon and was charged with 1,4-dihexyl-2,5-diiodobenzene[1] (441 mg, 885 μmol, 1.0 eq.), bis(triphenylphosphine) palladium(II) chloride (15.7 mg, 22.1 μmol, 2.5 mol%), CuI (6.78 mg, 35.4 μmol, 4 mol%) and the solvents THF/Et_3N (1:1, 100 mL). The mixture was degassed with argon for 15 min then *tert*-butyl(4-ethynylphenyl)sulfane[2] (450 mg, 2.36 mmol, 2.7 eq.) was added and the reaction mixture was stirred at 50 °C for 11 hours. After TLC confirmed full consumption of the starting material, the reaction was stopped. The mixture was concentrated, eluted with DCM (50 mL) and again concentrated on Silica. The crude product was then purified by flash column chromatography (cyclohexane) and automated GPC (chloroform) to yield **3** as a slightly yellow solid (395 mg, 634 μmol, 72%).

^{1}H NMR (400 MHz, $CDCl_3$, 22 °C) δ 7.55 – 7.50 (m, 4H), 7.49 – 7.45 (m, 4H), 7.37 (s, 2H), 2.85 – 2.77 (m, 4H), 1.76 – 1.63 (m, 4H), 1.47 – 1.37 (m, 4H), 1.37 – 1.27 (m, 26H), 0.92 – 0.83 (m, 6H).

^{13}C NMR (101 MHz, $CDCl_3$, 22 °C) δ 142.48, 137.42, 133.42, 132.52, 131.49, 123.97, 122.66, 93.62, 90.08, 46.67, 34.28, 31.91, 31.16, 30.78, 29.38, 22.78, 14.26.

HRMS (MALDI-TOF-MS): calc. for $[C_{42}H_{54}S_2]^+$ 622.3661; found 622.3645.

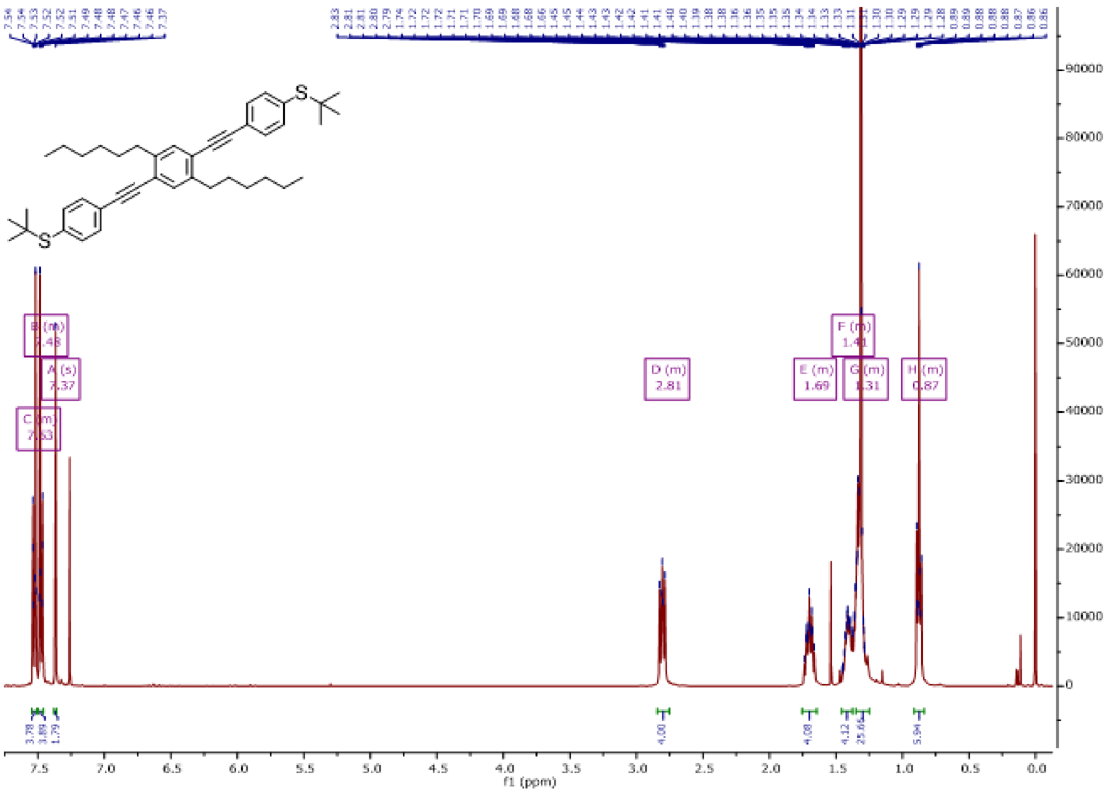

Figure S 5: **^{1}H-NMR** spectrum of **3** in $CDCl_3$.

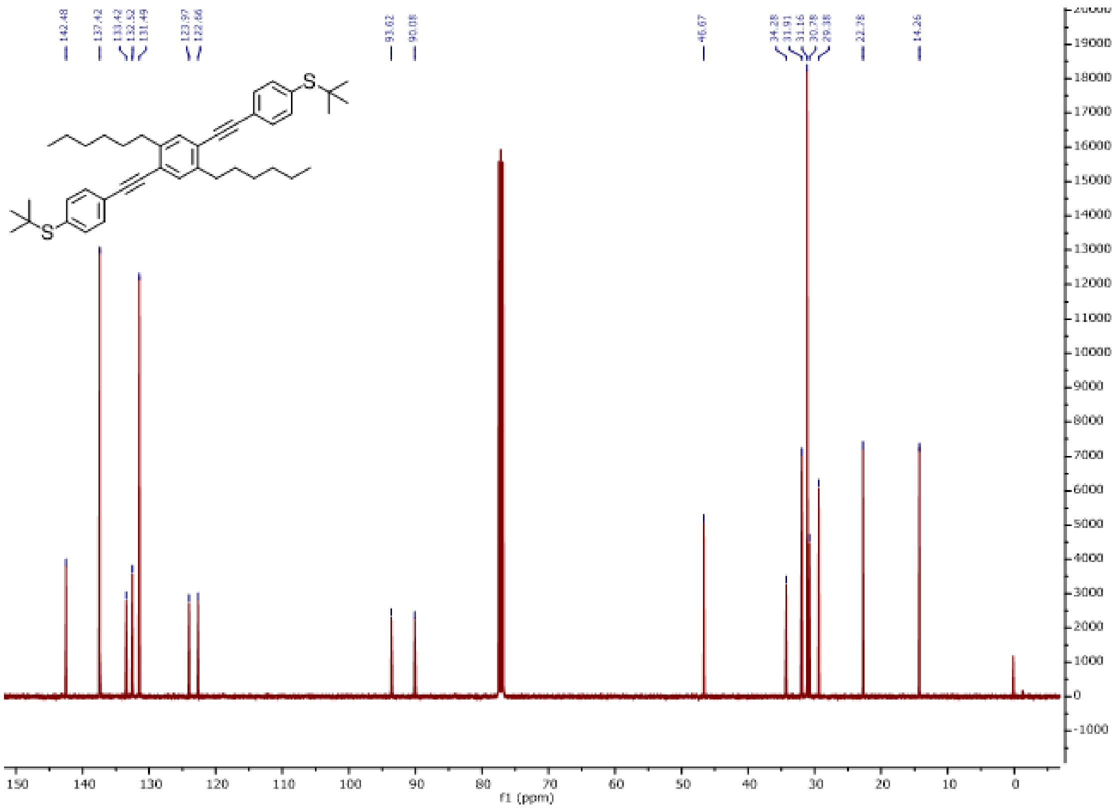

Figure S 6: **^{13}C{^{1}H}-NMR** spectrum of **3** in $CDCl_3$.

BSOL000448 Thomas Brandl/ - BRT615 - DCM - DCTB 1:10

ETH Eidgenössische Technische Hochschule Zürich
Swiss Federal Institute of Technology Zurich

Acquisition Parameter

Method:	MALDI_MS_POS_100-1000_2M_16AvScans			Acquisition Date:	13.05.2019 13:39:33
File Name:	D:\ETH\Data\BSOL0004xx\BSOL000448 _0_A14_000001.d			Operator:	
Source	Dual (MALDI/ESI)	Polarity	Positive	Nebulizer Gas	1.0 bar
Broadband Low Mass	77.0 m/z	n/a	n/a	Drying Gas Flow Rate	3.7 L/min
Broadband High Mass	1050.0 m/z	Laser Power	26.0 lp	Capillary	3500.0 V
No. of Cell Fills	1	n/a	n/a	Drying Gas	200.0 °C
Apodization	Full-Sine	Time of Flight to Detector	0.000 sec	Temperature	

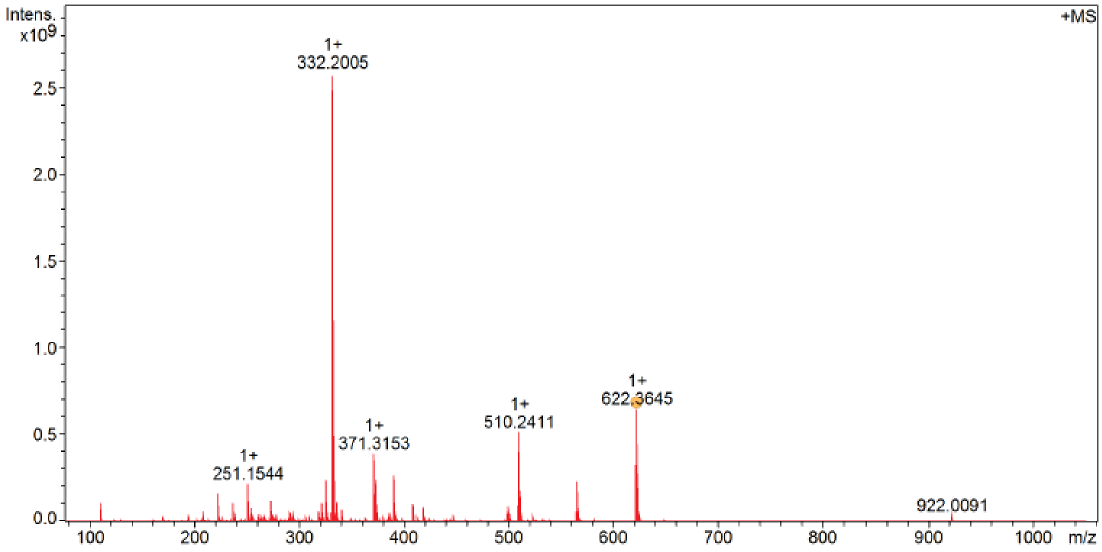

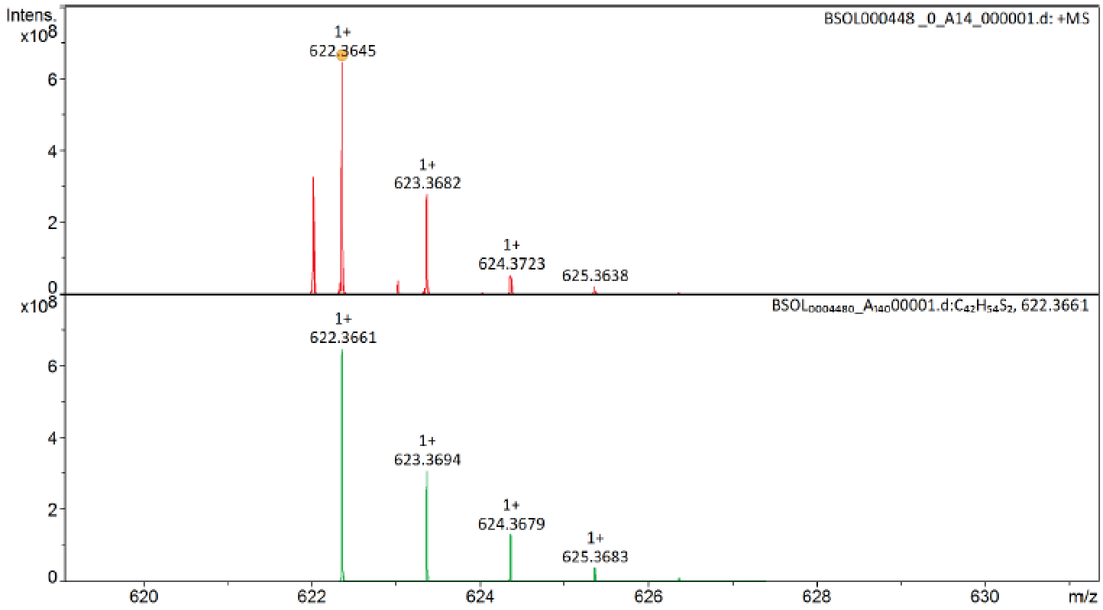

Bruker Daltonics solariX | ETH - MS-Service LOC - D-CHAB | Page 1 of 2

Figure S 7: **HRMS (MALDI, +)** spectrum of **3.**

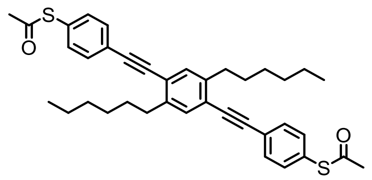

S,S'-(((2,5-dihexyl-1,4-phenylene)bis(ethyne-2,1-diyl))bis(4,1-phenylene)) diethanethioate (Hex-OPE3)

A 50 mL round bottomed flask was charged with (((2,5-dihexyl-1,4-phenylene)bis(ethyne-2,1-diyl))bis(4,1-phenylene))bis(*tert*-butylsulfane) (**3**, 50.0 mg, 80.3 μmol, 1.0 eq.), toluene (10 mL), acetonitrile (10 mL) and acetyl chloride (287 μL, 4.02 mmol, 50 eq.). To the stirred solution $Bi(OTf)_3$ (158 mg, 241 μmol, 3.0 eq.) was added. The reaction mixture was stirred at room temperature for 3 hours. After MALDI-TOF confirmed complete conversion of the starting material, water was added and the aqueous phase was extracted with DCM. The combined organic phases were dried over $MgSO_4$, filtered and concentrated under reduced pressure. The crude product was purified by automated GPC (chloroform) to yield **Hex-OPE3** as white solid (45.8 mg, 77.0 μmol, 96%).

^{1}H NMR (500 MHz, $CDCl_3$, 25 °C) δ 7.56 – 7.53 (m, 4H), 7.43 – 7.39 (m, 4H), 7.37 (s, 2H), 2.83 – 2.76 (m, 4H), 2.44 (s, 6H), 1.73 – 1.65 (m, 4H), 1.45 – 1.37 (m, 4H), 1.36 – 1.30 (m, 8H), 0.91 – 0.85 (m, 6H).

^{13}C NMR (126 MHz, $CDCl_3$, 25 °C) δ 193.62, 142.58, 134.43, 132.58, 132.18, 128.18, 124.87, 122.61, 93.39, 90.24, 34.30, 31.91, 30.80, 30.45, 29.40, 22.78, 14.26.

HRMS (MALDI-TOF-MS): calc. for $[C_{38}H_{42}O_2S_2]^+$ 594.2621; found 594.2623.

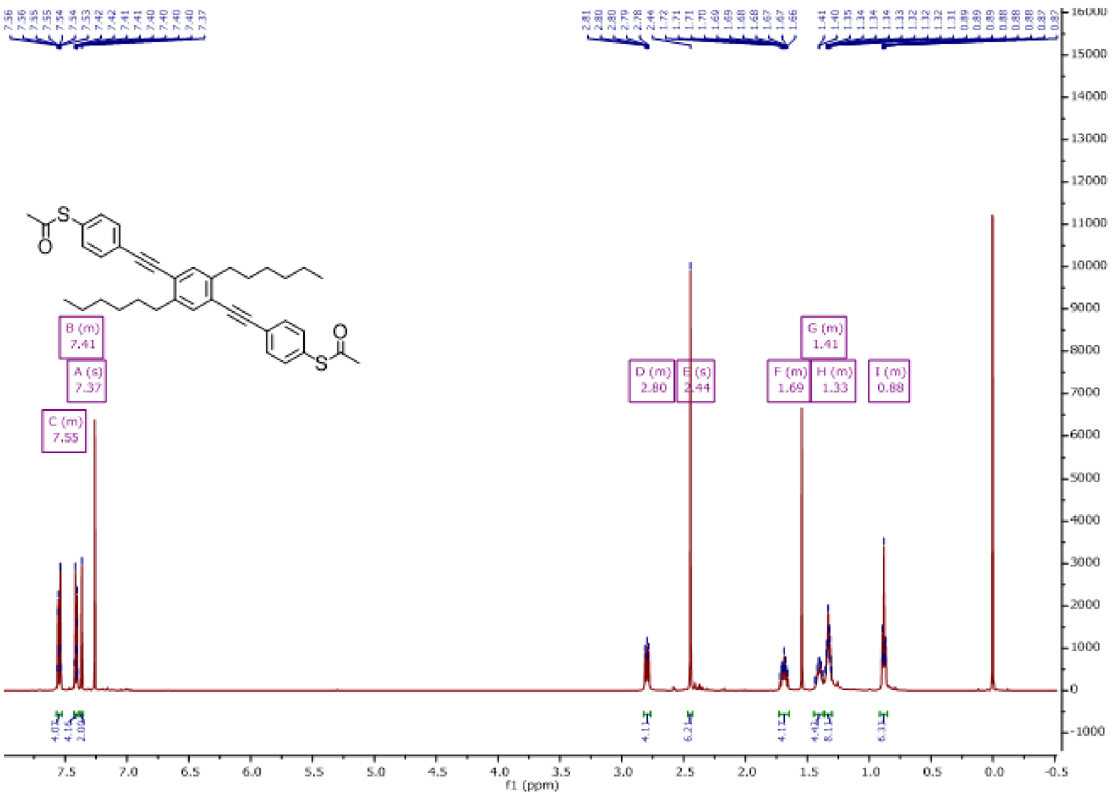

Figure S 8: **^{1}H-NMR** spectrum of **Hex-OPE3** in $CDCl_3$.

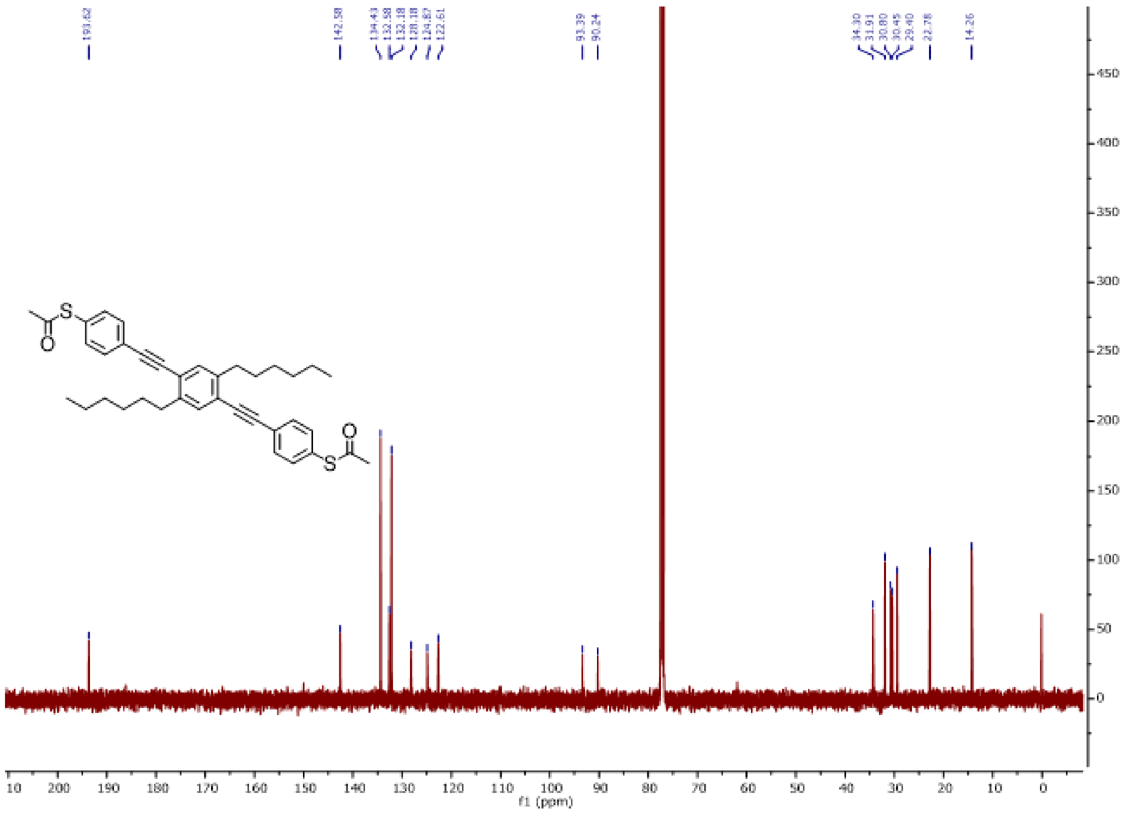

Figure S 9: **$^{13}C\{^1H\}$-NMR** spectrum of **Hex-OPE3** in $CDCl_3$.

BSOL000449 Thomas Brandl/ - BRT617 - DCM - DCTB 1:10

ETH Eidgenössische Technische Hochschule Zürich
Swiss Federal Institute of Technology Zurich

Acquisition Parameter

Method:	MALDI_MS_POS_100-1000_2M_16AvScans			Acquisition Date:	13.05.2019 13:46:17
File Name:	D:\ETH\Data\BSOL0004xx\BSOL000449 _0_A21_000001.d			Operator:	
Source	Dual (MALDI/ESI)	Polarity	Positive	Nebulizer Gas	1.0 bar
Broadband Low Mass	77.0 m/z	n/a	n/a	Drying Gas Flow Rate	3.7 L/min
Broadband High Mass	1050.0 m/z	Laser Power	28.4 lp	Capillary	3500.0 V
No. of Cell Fills	1	n/a	n/a	Drying Gas	200.0 °C
Apodization	Full-Sine	Time of Flight to Detector	0.000 sec	Temperature	

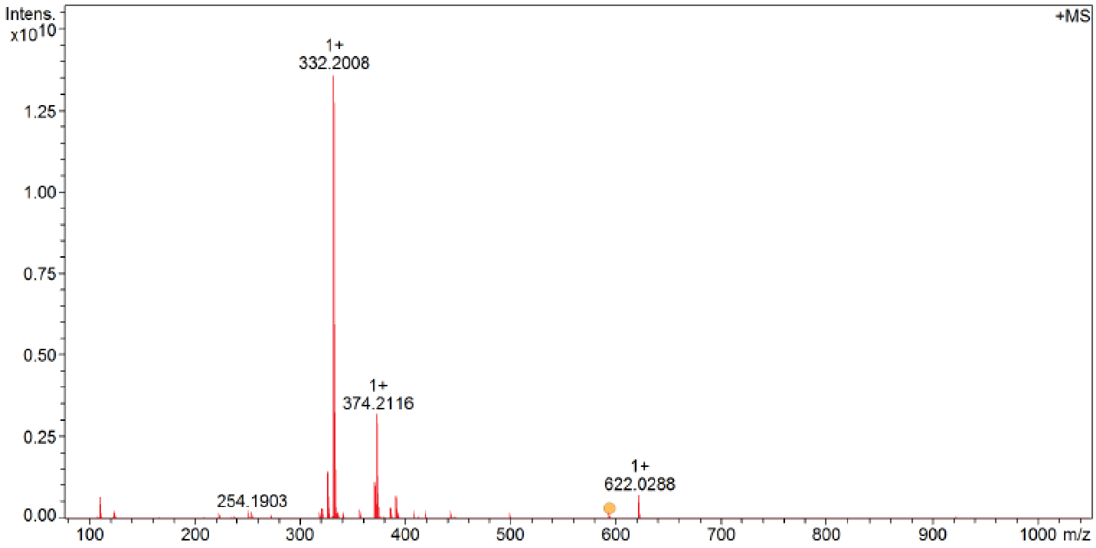

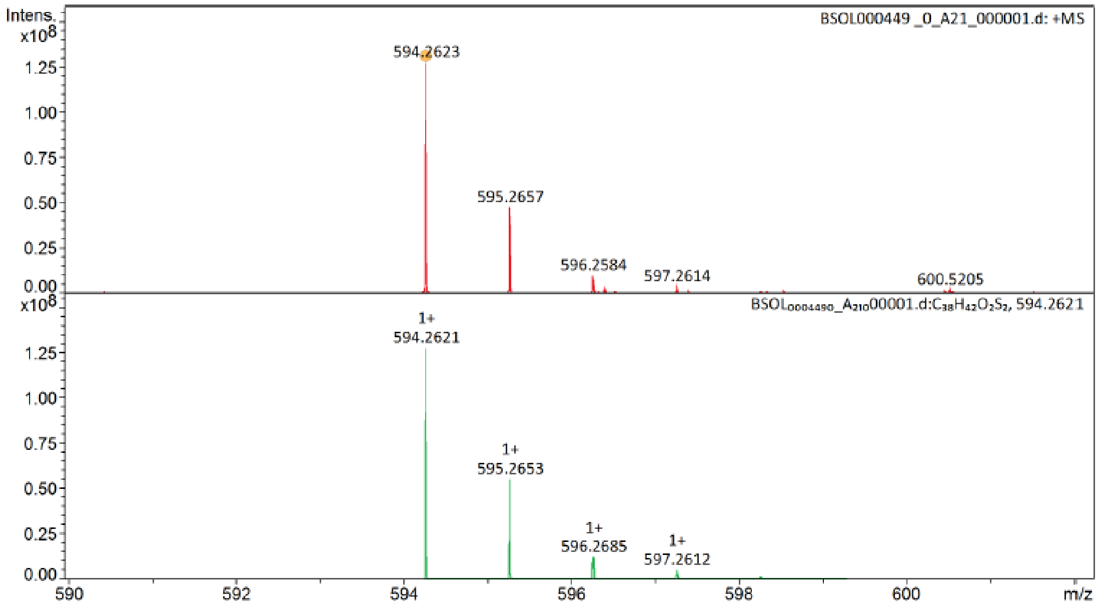

Figure S 10: **HRMS (MALDI, +)** spectrum of **Hex-OPE3.**

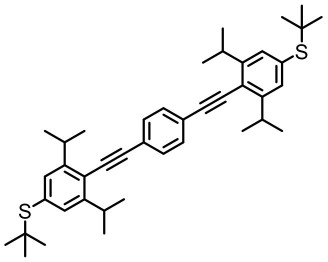

1,4-bis((4-(*tert*-butylthio)-2,6-diisopropylphenyl)ethynyl) benzene (4)

Tert-butyl(4-ethynyl-3,5-diisopropylphenyl)sulfane was synthesized according to literature. A 50 mL round bottom flask was purged with argon and was charged with 1,4-diiodobenzene (15.0 mg, 45.5 µmol, 1.0 eq.), bis(triphenylphosphine) palladium(II) chloride (806 µg, 1.14 µmol, 2.5 mol%), CuI (348 µg, 1.82 µmol, 4 mol%) and the solvents THF/Et_3N (1:1, 30 mL). The mixture was degassed with argon for 15 min then *tert*-butyl(4-ethynyl-3,5-diisopropylphenyl)sulfane[3] (31.2 mg, 114 µmol, 2.5 eq.) was added and the reaction mixture was stirred at 50 °C for 12 hours. After TLC confirmed full consumption of the starting material, the reaction was stopped. The mixture was concentrated, eluted with DCM (50 ml) and again concentrated on Silica. The crude product was then purified by flash column chromatography (cyclohexane) and automated GPC (chloroform) to yield **4** as white solid (11.9 mg, 19.0 µmol, 42%).

^{1}H NMR (400 MHz, $CDCl_3$, 22 °C) δ 7.52 (s, 4H), 7.31 (s, 4H), 3.58 (hept, *J* = 6.8 Hz, 4H), 1.32 (s, 24H), 1.31 (d, *J* = 4.1 Hz, 18H).

^{13}C NMR (126 MHz, $CDCl_3$, 25 °C) δ 150.97, 133.40, 131.47, 131.44, 123.63, 121.25, 98.42, 88.40, 46.38, 31.94, 31.17, 23.34.

HRMS (MALDI-TOF-MS): calc. for $[C_{42}H_{54}S_2]^+$ 622.3661; found 622.3662.

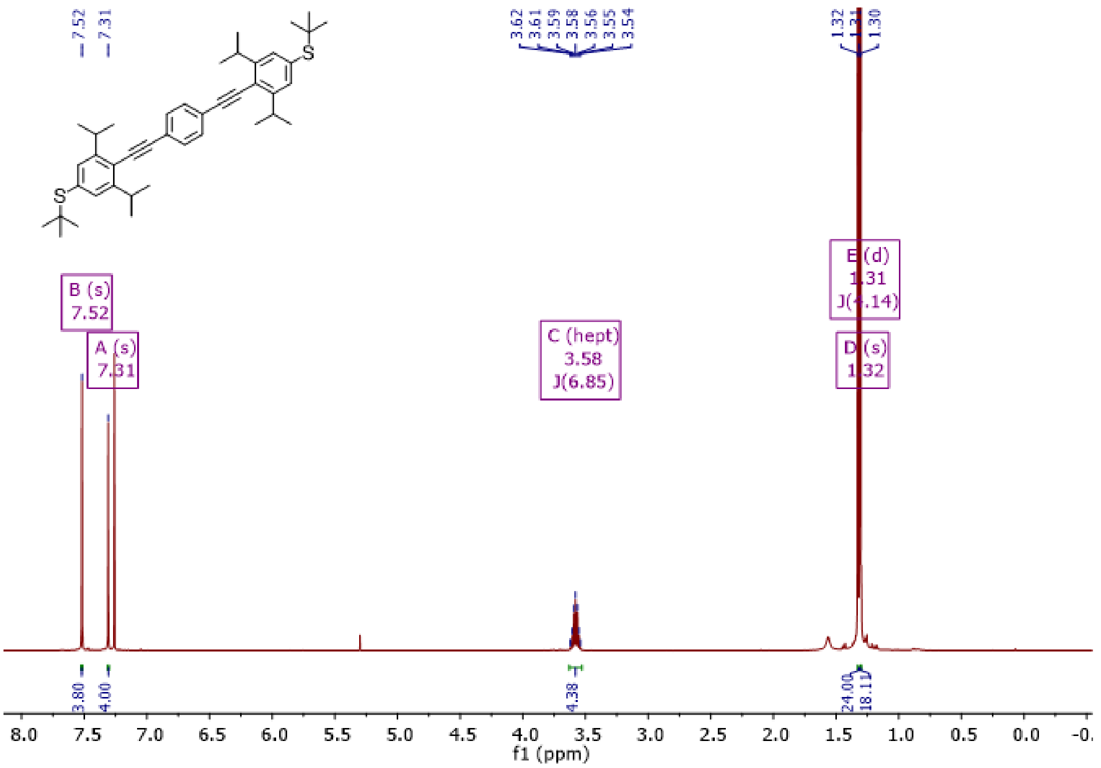

Figure S 11: **^{1}H-NMR** spectrum of **4** in $CDCl_3$.

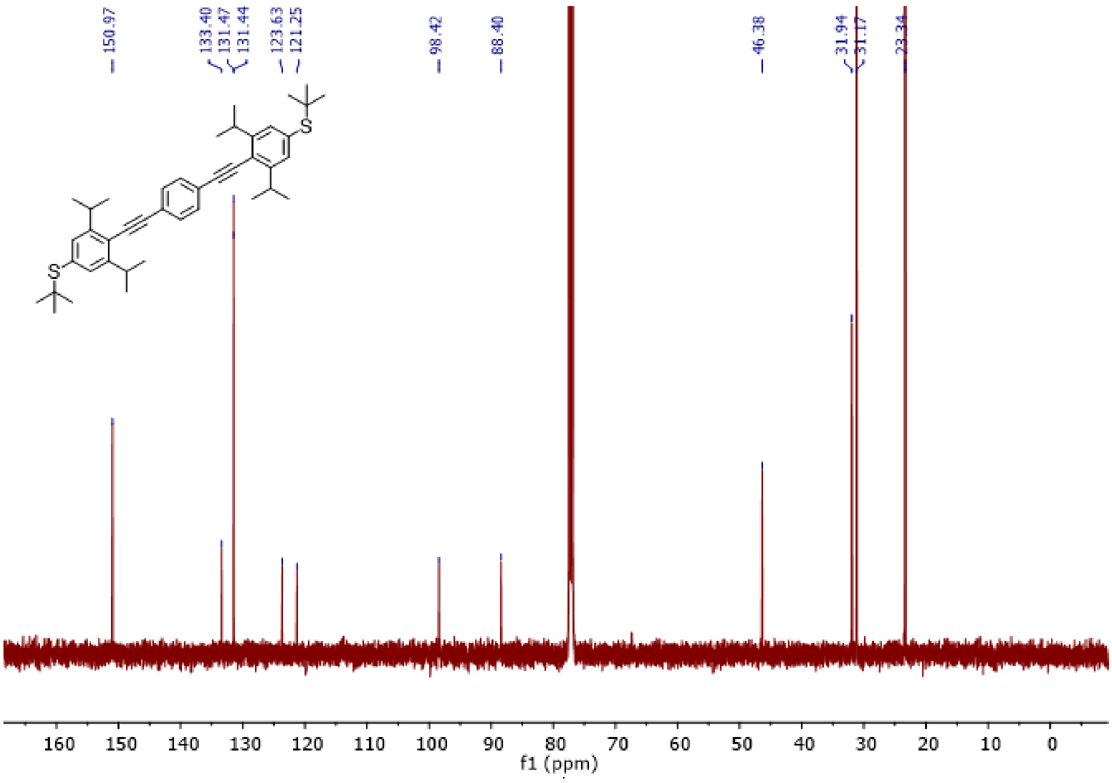

Figure S 12: **^{13}C{^{1}H}-NMR** spectrum of **4** in $CDCl_3$.

FT13279 Thomas Brandl/Mayor - BRT479 - DCM / DCTB Mix 1:10

ETH Eidgenössische Technische Hochschule Zürich
Swiss Federal Institute of Technology Zurich

Acquisition Parameter

Method:	MALDI_MS_POS_100-1000_2M_16AvScans			Acquisition Date:	03.07.2018 10:35:50
File Name:	D:\ETH\Data\FT132xx\FT13279_0_A11_000001.d			Operator:	Louis Bertschi
Source	Dual (MALDI/ESI)	Polarity	Positive	Nebulizer Gas	1.0 bar
Broadband Low Mass	77.0 m/z	n/a	n/a	Drying Gas Flow Rate	3.7 L/min
Broadband High Mass	1050.0 m/z	Laser Power	33.0 lp	Capillary	4500.0 V
No. of Cell Fills	1	n/a	n/a	Drying Gas	200.0 °C
Apodization	Full-Sine	Time of Flight to Detector	0.001 sec	Temperature	

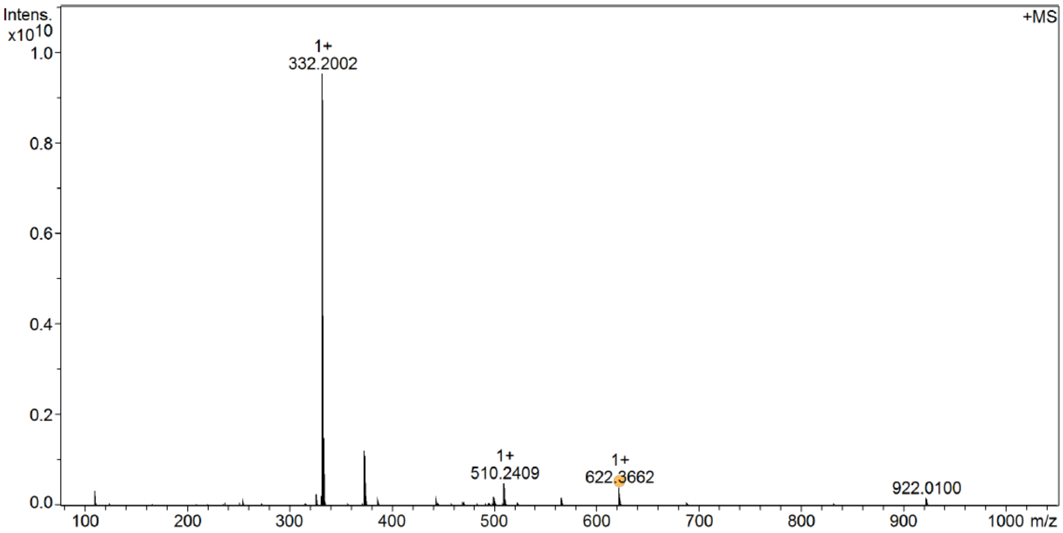

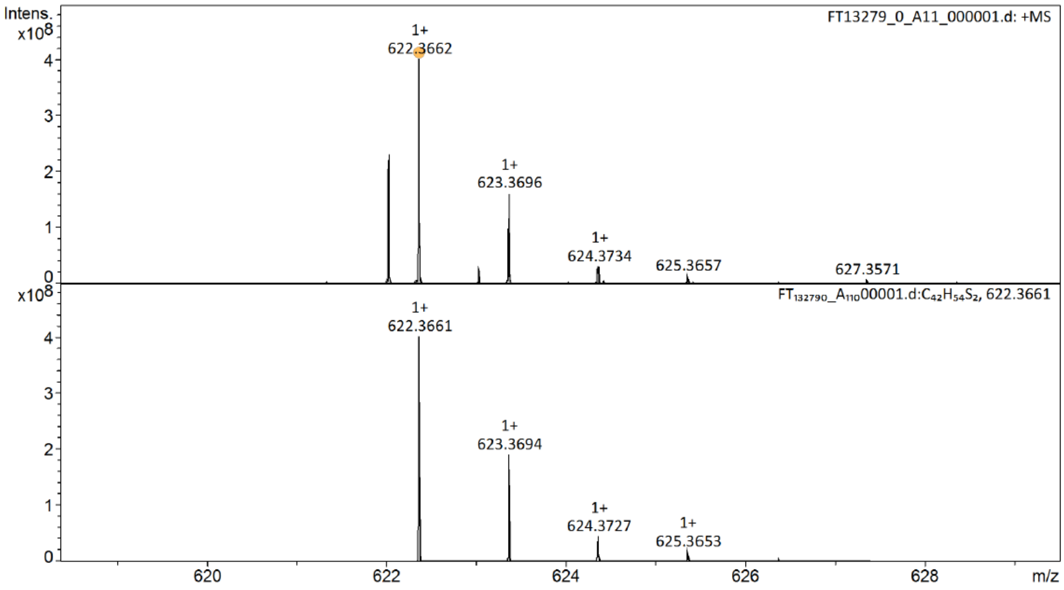

Figure S 13: **HRMS (MALDI, +)** spectrum of **4.**

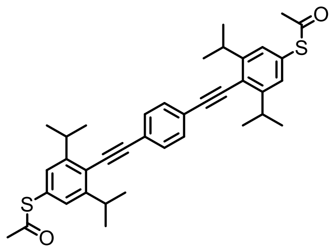

S,S'-((1,4-phenylenebis(ethyne-2,1-diyl)bis(3,5-diisopropyl-4,1-phenylene))diethanethioate (^{i}Pr-OPE3)

A 50 mL round bottomed flask was charged with 1,4-bis((4-(*tert*-butylthio)-2,6-diisopropylphenyl)ethynyl)benzene (**4**, 10.5 mg, 16.9 µmol, 1.0 eq.), toluene (10 mL), acetonitrile (10 mL) and acetyl chloride (60.3 µL, 845 µmol, 50 eq.). To the stirred solution $Bi(OTf)_3$ (33.3 mg, 50.7 µmol, 3.0 eq) was added. The reaction mixture was stirred at room temperature for 3 hours. After MALDI-TOF confirmed complete conversion of the starting material, water was added and the aqueous phase was extracted with DCM. The combined organic phases were dried over $MgSO_4$, filtered and concentrated under reduced pressure. The crude product was purified by automated GPC (chloroform) to yield **^{i}Pr-OPE3** as white solid (10.0 mg, 17.0 µmol, 100%).

^{1}H NMR (500 MHz, CD_2Cl_2, 25 °C) δ 7.56 (d, *J* = 1.1 Hz, 4H), 7.22 (s, 2H), 3.62 (hept, *J* = 6.7 Hz, 3H), 2.42 (d, *J* = 2.5 Hz, 5H), 1.32 (d, *J* = 6.8 Hz, 20H).

^{13}C NMR (126 MHz, CD_2Cl_2, 25 °C) δ 194.26, 152.26, 131.90, 129.28, 128.99, 124.02, 122.50, 99.05, 88.50, 32.55, 30.67, 23.41.

HRMS (MALDI-TOF-MS): calc. for $[C_{38}H_{42}O_2S_2]^+$ 594.2621; found 594.2615.

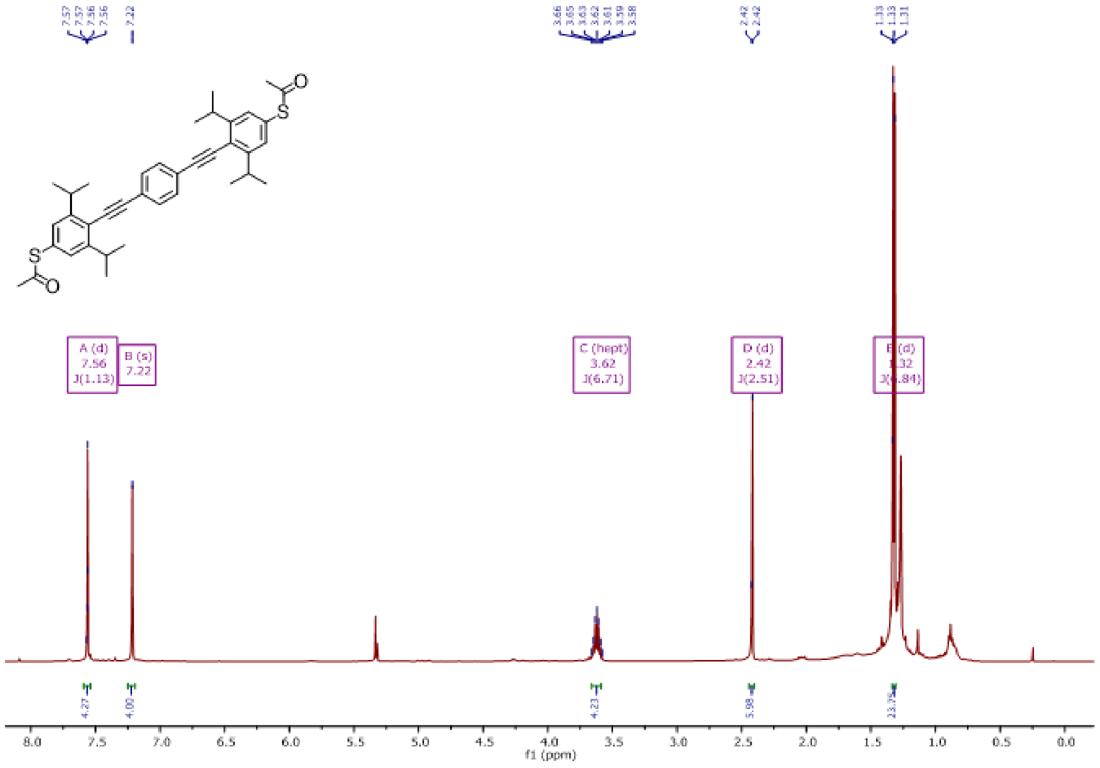

Figure S 14: **^{1}H-NMR** spectrum of **^{i}Pr-OPE3** in $CDCl_3$.

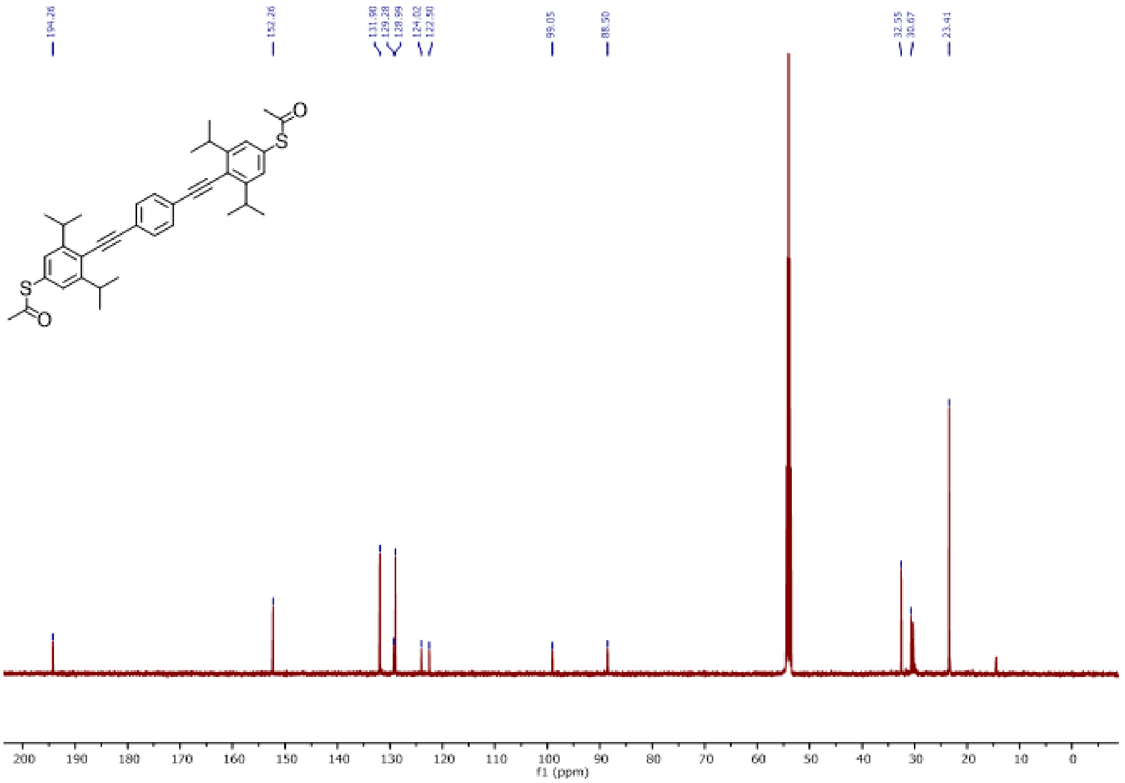

Figure S 15: **^{13}C{^{1}H}-NMR** spectrum of **^{i}Pr-OPE3** in $CDCl_3$.

FT13280 Thomas Brandl/Mayor - BRT480 - DCM / DCTB Mix 1:10

ETH
Eidgenössische Technische Hochschule Zürich
Swiss Federal Institute of Technology Zurich

Acquisition Parameter

Method:	MALDI_MS_POS_100-1000_2M_16AvScans	Acquisition Date:	03.07.2018 10:36:35
File Name:	D:\ETH\Data\FT132xx\FT13280_0_A14_000001.d	Operator:	Louis Bertschi

Source	Dual (MALDI/ESI)	Polarity	Positive	Nebulizer Gas	1.0 bar
Broadband Low Mass	77.0 m/z	n/a	n/a	Drying Gas Flow Rate	3.7 L/min
Broadband High Mass	1050.0 m/z	Laser Power	33.0 lp	Capillary	4500.0 V
No. of Cell Fills	1	n/a	n/a	Drying Gas	200.0 °C
Apodization	Full-Sine	Time of Flight to Detector	0.001 sec	Temperature	

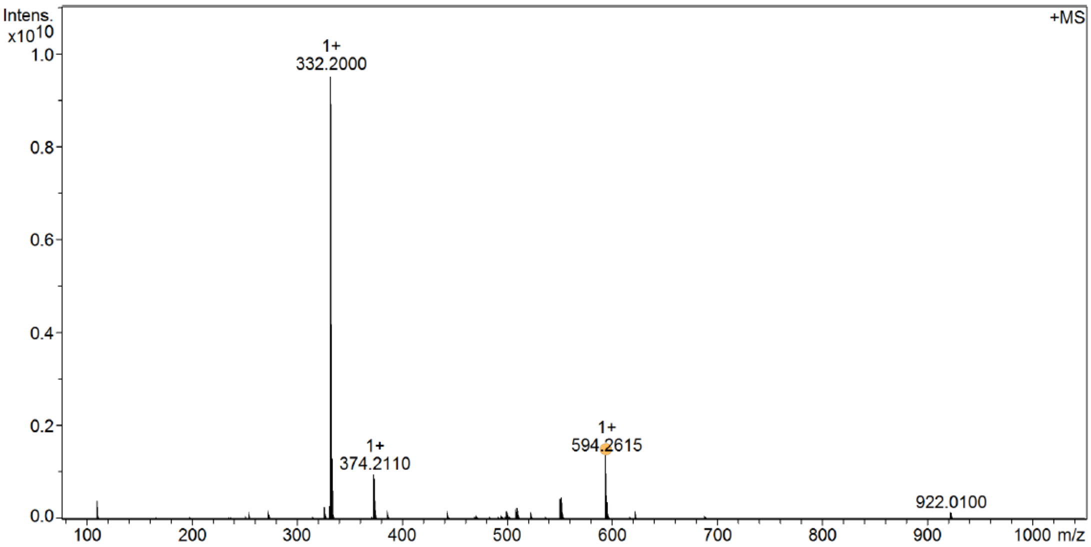

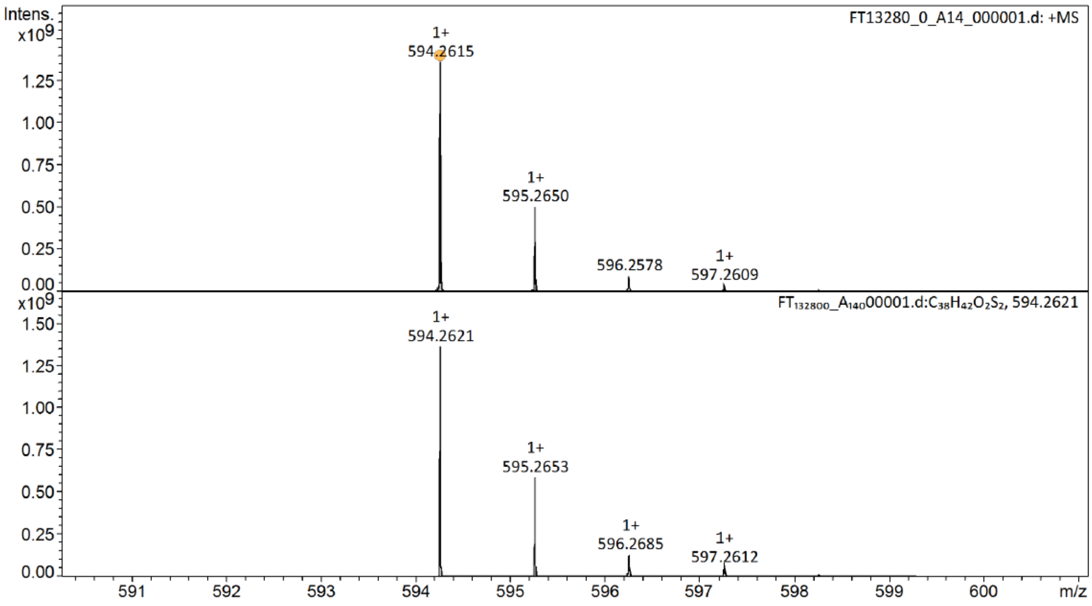

Figure S 16: **HRMS (MALDI, +)** spectrum of ^{i}**Pr-OPE3.**

S2 Mechanically Controllable Break Junction (MCBJ) Measurements

In this section we report additional information, plots and tables supporting the discussion on the MCBJ measurements.

The OPE3 measurements were performed with a piezo actuation speed of 50 V/s, corresponding to about 1 nm/s of electrode speed. The breaking process proceeds for 200 – 250 V (about 4 – 5 nm) after the conductance drops below 10 G_0. Then the junctions are merged back together until their conductance reaches 30 G_0, and a new breaking trace is started.

S2.1 Clustering Parameters

As mentioned in the main text, we employ an approach based on *kmeans++* described in ref. [4]. For defining the feature space, we used a 30x30 bins two-dimensional (2D) conductance-displacement histogram using the range -2.5 to -6 log(G/G_0) in conductance and 0 to 3.5 nm in displacement. Additionally, we appended the one-dimensional (1D) conductance histogram constructed in the range -2.5 to -6 log(G/G_0) with 100 bins. The exact same clustering settings were used for all molecules.

S2.2 Supplementary Plots and Tables

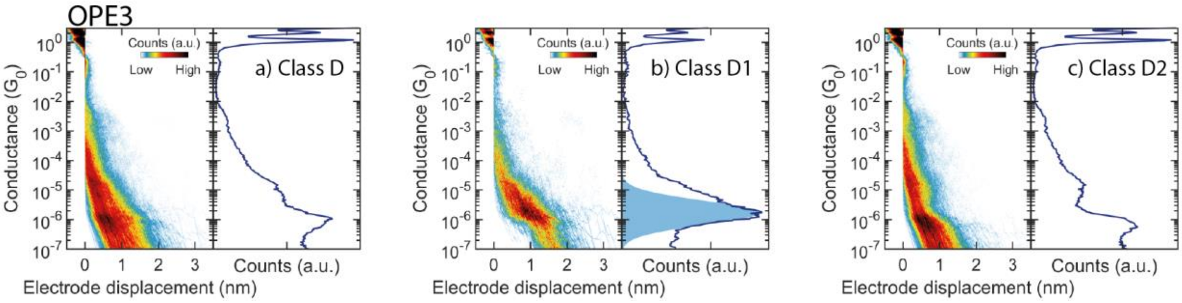

Figure S 17: 2D conductance-displacement (*left panels*) and 1D conductance histograms (*right panels*) of a) Class D of **OPE3**, and of the further splitting into b) Class D1 and c) Class D2.

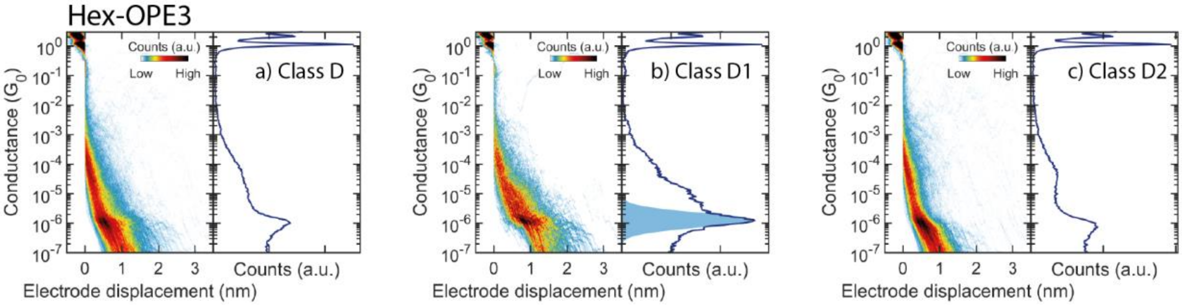

Figure S 18: 2D conductance-displacement (*left panels*) and 1D conductance histograms (*right panels*) of a) Class D of **Hex-OPE3**, and of the further splitting into b) Class D1 and c) Class D2.

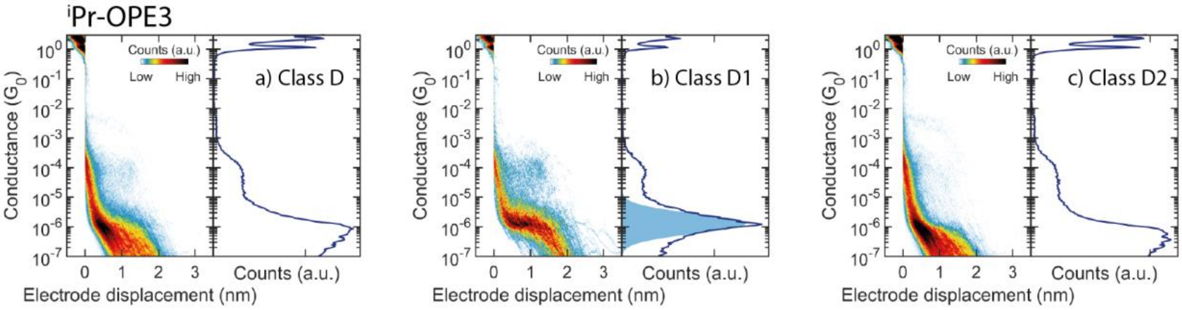

Figure S 19: 2D conductance-displacement (*left panels*) and 1D conductance histograms (*right panels*) of a) Class D of **^{i}Pr-OPE3**, and of the further splitting into b) Class D1 and c) Class D2.

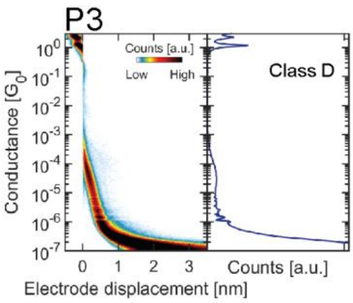

Figure S 20: 2D conductance-displacement (*left panel*) and 1D conductance histogram (*right panel*) of Class D of **P3**.

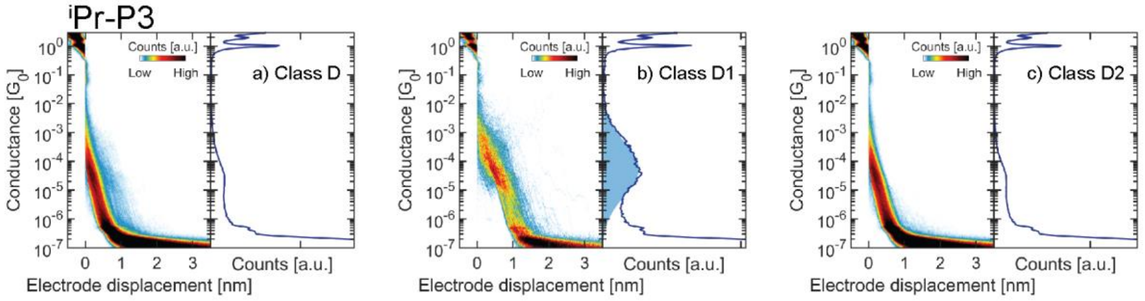

Figure S 21: 2D conductance-displacement (*left panels*) and 1D conductance histograms (*right panels*) of a) Class D of **iPr-P3**, and of the further splitting into b) Class D1 and c) Class D2.

Table S 1: Table reporting for each molecule the number of traces measured, the conductance, full-width half-maximum (FWHM), length, relative yield (number of traces in the class / number of molecular traces) and total yield (number of traces in the class / total number of traces).

Molecule	N traces	Class	Conductance (G_0)	FWHM (decades)	Length (nm)	Relative Yield (%)	Total Yield (%)
**OPE3**	5000	A	$2.6 \cdot 10^{-4}$	0.8	1.4	23	14
		B	$1.2 \cdot 10^{-4}$	0.8	1.5	32	19
		C	$4.3 \cdot 10^{-5}$	1.2	1.2	28	17
		D	//	//	//	//	50
		D1	$1.7 \cdot 10^{-6}$	1.4	1.4	17	10
		D2	//	//	//	//	40
**Hex-OPE3**	6417	A	$3.0 \cdot 10^{-4}$	0.9	1.5	51	39
		B	$1.1 \cdot 10^{-4}$	0.9	1.4	27	21
		C	$3.4 \cdot 10^{-5}$	1.0	1.0	11	8
		D	//	//	//	//	32
		D1	$1.3 \cdot 10^{-6}$	1.6	1.3	11	8
		D2	//	//	//	//	24
**^{i}Pr-OPE3**	7297	A	$4.3 \cdot 10^{-4}$	1.1	1.5	57	48
		B	$1.2 \cdot 10^{-4}$	1.0	1.4	31	26
		C	$2.8 \cdot 10^{-5}$	1.5	1.1	7	6
		D	//	//	//	//	20
		D1	$1.3 \cdot 10^{-6}$	1.0	1.6	5	4
		D2	//	//	//	//	16
**P3**	9999	A’	$3.0 \cdot 10^{-4}$	0.8	2.5	77	10
		B’	$1.3 \cdot 10^{-5}$	1.3	1.9	15	2
		C’	$1.4 \cdot 10^{-6}$	0.7	1.1	8	1
		D’	//	//	//	//	87
**^{i}Pr-P3**	10000	A’	$3.3 \cdot 10^{-4}$	1.1	2.1	43	16
		B’	$7.1 \cdot 10^{-5}$	1.1	2.5	38	14
		C’	$4.6 \cdot 10^{-6}$	1.5	1.8	3	1
		D’	//	//	//	//	69
		D1’	$5.4 \cdot 10^{-5}$	2.6	1.0	16	6
		D2	//	//	//	//	63

S3 References

1. N. M. Jenny, H. Wang, M. Neuburger, H. Fuchs, L. Chi, M. Mayor, *Eur. J. Org. Chem.* **2012**, *2012*, 2738–2747.
2. A. Błaszczyk, M. Elbing, M. Mayor, *Org. Biomol. Chem.* **2004**, *2*, 2722–2724.
3. T. Brandl, M. E. Abbassi, D. Stefani, R. Frisenda, G. D. Harzmann, H. S. J. van der Zant, M. Mayor, *Eur. J. Org. Chem.* **2019**, *2019*, 5334–5343.
4. D. Cabosart, M. El Abbassi, D. Stefani, R. Frisenda, M. Calame, H.S.J. van der Zant, M.L. Perrin, *Appl. Phys. Lett.* **2019**, 114, 143102.
